# Formulation Strategies for Immunomodulatory Natural Products in 3D Tumor Spheroids and Organoids: Current Challenges and Emerging Solutions

**DOI:** 10.3390/pharmaceutics17101258

**Published:** 2025-09-25

**Authors:** Chang-Eui Hong, Su-Yun Lyu

**Affiliations:** 1 College of Pharmacy, Sunchon National University, Suncheon 57922, Republic of Korea; 2 Smart Beautytech Research Institute, Sunchon National University, Suncheon 57922, Republic of Korea; 3 Research Institute of Life and Pharmaceutical Sciences, Sunchon National University, Suncheon 57922, Republic of Korea

**Keywords:** 3D tumor models, formulation strategies, immunomodulation, natural products, penetration–activity trade-off

## Abstract

**Background/Objectives:** Natural products exhibit significant immunomodulatory potential but face severe efficacy loss in three-dimensional (3D) tumor models. This review comprehensively examines the penetration–activity trade-off and proposes integrated strategies for developing effective natural product-based cancer immunotherapies. **Methods:** We analyzed formulation strategies across three natural product categories (hydrophobic, macromolecular, stability-sensitive), evaluating penetration enhancement versus activity preservation in spheroids, organoids, and advanced 3D platforms. **Results:** Tumor spheroids present formidable barriers: dense extracellular matrix (33-fold increased fibronectin), pH gradients (7.4 → 6.5), and extreme cell density (6 × 10^7^ cells/cm^3^). While nanoparticles, liposomes, and cyclodextrins achieve 3–20-fold penetration improvements, biological activity frequently declines through conformational changes, incomplete release (10–75%), and surface modification interference. Critically, immune cells remain peripheral (30–50 μm), questioning deep penetration pursuit. Patient-derived organoids display 68% predictive accuracy, while emerging vascularized models unveil additional complexity. Food and Drug Administration (FDA) Modernization Act 2.0 enables regulatory acceptance of these advanced models. **Conclusions:** Effective therapeutic outcomes depend on maintaining immunomodulatory activity in peripherally-located immune cell populations rather than achieving maximum tissue penetration depth. Our five-stage evaluation framework and standardization protocols guide development. Future priorities include artificial intelligence-driven optimization, personalized formulation strategies, and integration of multi-organ platforms to bridge the critical gap between enhanced delivery and therapeutic efficacy.

## 1. Introduction

Cancer immunotherapy has revolutionized oncology treatment, yet clinical success remains limited by inadequate preclinical models that fail to predict human responses [1,2]. Natural products, representing millennia of therapeutic wisdom, offer unique immunomodulatory properties through diverse mechanisms—from checkpoint modulation to macrophage reprogramming [3,4,5]. However, their therapeutic potential encounters formidable barriers when transitioning from simplified culture systems to physiologically relevant three-dimensional (3D) tumor models [5,6,7].

The journey from bench to bedside for immunomodulatory natural products reveals a fundamental challenge: compounds with remarkable efficacy in conventional assays often fail dramatically in complex tumor microenvironments. Curcumin exemplifies this paradox—while exhibiting potent programmed death-ligand 1 (PD-L1) downregulation in monolayer cultures [8,9], its limited penetration into the dense multicellular architecture of tumor spheroids compromises its therapeutic efficacy [10,11,12]. This penetration-efficacy disconnect has been observed across diverse natural product classes—including terpenoids, alkaloids, flavonoids, and polysaccharides—each facing unique physicochemical obstacles related to their molecular properties [12].

3D tumor models have emerged as essential platforms bridging the translational gap between Petri dishes and patients [13,14,15]. Unlike their two-dimensional (2D) counterparts, these systems recapitulate critical features of human tumors: heterogeneous cell populations, oxygen gradients, drug penetration barriers, and complex extracellular matrix architectures [16,17]. Spheroids, organoids, and scaffold-based constructs each offer distinct advantages for evaluating natural product behavior in environments mimicking clinical reality [18,19,20].

The physicochemical diversity of immunomodulatory natural products necessitates tailored formulation approaches. Hydrophobic compounds such as resveratrol face aggregation and protein binding that severely restrict bioavailability [21,22,23]. Macromolecular entities including medicinal mushroom β-glucans encounter size-dependent exclusion from dense tumor matrices [24,25,26]. pH-sensitive molecules like anthocyanins undergo rapid degradation in acidic microenvironments, potentially losing activity before reaching target immune populations [27,28]. Each category demands innovative solutions balancing enhanced delivery with preserved biological function.

Recent technological convergence creates unprecedented opportunities for addressing these challenges. Advanced formulation strategies—from stimuli-responsive nanocarriers to cyclodextrin complexation—provide remarkable penetration improvements [29,30,31,32]. Simultaneously, sophisticated 3D culture platforms now incorporate vascular networks, immune cell populations, and real-time monitoring capabilities [32,33,34]. The intersection of these technologies with regulatory support through Food and Drug Administration (FDA) Modernization Act 2.0 positions the field for transformative advances [13,34,35,36,37].

This review provides the first systematic analysis of the penetration–activity trade-off for natural product formulations in 3D tumor models, a critical gap that has hindered clinical translation. Unlike previous reviews focusing solely on penetration enhancement or biological activity, we uniquely integrate both perspectives. Our specific objectives are to: (1) quantify the extent of activity loss despite penetration improvements, (2) map the spatial distribution of immune cells and its implications for formulation design, (3) establish compound-specific strategies for three natural product categories (hydrophobic, macromolecular, stability-sensitive), and (4) propose a five-stage evaluation framework bridging formulation optimization with clinical implementation. By addressing the fundamental disconnect between pharmaceutical optimization and biological efficacy, this review guides the development of natural product-based immunotherapies that maintain therapeutic activity while achieving effective tumor penetration.

This comprehensive narrative review synthesizes key literature, selected based on mechanistic relevance and innovation in formulation strategies rather than systematic database searches, to develop an integrated framework applicable across diverse natural product categories and 3D model platforms.

To contextualize the scope and timeliness of this narrative review, we analyzed the research landscape through both our curated references and database searches. Our 531 references reveal a clear evolution in the field: foundational studies (2015–2019) primarily focused on characterizing natural product properties, while recent literature (2020–2025) increasingly addresses their integration with advanced 3D culture methodologies. A complementary search in PubMed and Web of Science databases (accessed on 10 July 2025) using terms (‘spheroid’ OR ‘organoid’) AND (‘drug screening’ OR ‘natural product’ OR ‘phytochemical’) yielded 5,673 publications, showing a 3.6-fold increase between 2015–2019 and 2020–2025, with notable acceleration following the FDA Modernization Act 2.0.

The bibliographic analysis reveals four dominant research clusters that emerged sequentially but now converge: First, penetration enhancement strategies in 3D systems evolved from simple size optimization to sophisticated multi-stage delivery systems (Section 3.4). Second, microfluidic 3D platforms progressed from basic perfusion models to integrated organ-on-chip systems (Section 5.1.3). Third, patient-derived organoid technology advanced from proof-of-concept studies to clinical validation with 68% positive predictive value (Section 5.1.2). Fourth, immune cell integration in 3D models expanded from simple co-cultures to complex spatial mapping of tumor-immune interactions (Section 4.2).

This global collaborative effort, reflected in our reference collection spanning multiple countries and institutions, underscores the interdisciplinary nature of this field. The convergence of these previously disparate research streams—now evident in integrated studies combining advanced formulations with sophisticated 3D models—provides the foundation for our conceptual framework addressing the penetration–activity trade-off.

## 2. Penetration Barriers in 3D Tumor Models

The transition from 2D to 3D culture systems reveals fundamental barriers that natural products must overcome to achieve therapeutic efficacy. Understanding these barriers at molecular, cellular, and tissue levels provides essential foundation for rational formulation design.

### 2.1. Microenvironmental Characteristics of 3D Models

#### 2.1.1. Physical Architecture and Matrix Composition

The expression of extracellular matrix components in spheroids shows dramatic increases, with fibronectin levels elevated up to 33-fold compared to 2D cultures, while collagen I, collagen IV, and laminin also demonstrate significant upregulation. This upregulation results from enhanced collagen I and IV deposition, with fibronectin and laminin creating additional structural complexity. The resulting dense extracellular matrix (ECM) network, enriched with these upregulated components, creates a complex microenvironment that influences drug penetration and cellular behavior [38,39,40].

As spheroids mature, this enhanced ECM deposition drives progressive cellular compaction through cell-ECM and cell–cell interactions [41,42]. The mechanical forces generated by the contracting ECM network compress cells together [41], reducing intercellular spaces and creating a densely packed structure that mimics solid tumor architecture [7,43,44].

Cell packing density further compounds penetration challenges, reaching 6 × 10^7^ cells/cm^3^ in mature spheroids compared to 1.8–3.6 × 10^6^ cells/cm^3^ in confluent monolayers [45,46]. This increased density reduces interstitial space in core regions [47], resulting in tortuous diffusion pathways that significantly retard drug penetration [48].

The structural heterogeneity within spheroids generates distinct zones with varying penetration characteristics. Peripheral regions maintain relatively higher porosity due to active proliferation and looser cell packing [47], while core regions exhibit minimal interstitial space due to compression and reduced proliferation [47,49]. This radial gradient in structural density establishes a formidable barrier to drug delivery, requiring molecules to navigate increasingly constricted pathways as they move toward the spheroid center [48]. These structural barriers work in concert with chemical gradients to form a complex microenvironment that challenges drug delivery.

#### 2.1.2. Chemical Gradient Formation

The restricted mass transport in 3D models produces steep chemical gradients that profoundly impact natural product behavior. Unlike monolayer cultures where cells experience uniform conditions, spheroids develop characteristic microenvironmental zones that challenge drug delivery and stability (Table 1) [50,51].

Oxygen gradients represent the most critical factor, with hypoxic cores driving metabolic reprogramming toward glycolysis [50,51]. This metabolic shift not only alters cellular drug response but also produces metabolic byproducts that further modify the microenvironment [52,53]. The resulting lactate accumulation leads to an acidic milieu that can reach levels comparable to those found in solid tumors, establishing conditions that markedly affect the stability and activity of pH-sensitive natural products [54,55,56].

These gradients establish phenotypically different cell populations with varying drug sensitivities. The proliferative periphery maintains oxidative metabolism and active drug uptake mechanisms, while the quiescent intermediate zone exhibits reduced metabolic activity and altered drug response patterns [52,53]. The necrotic core, characterized by severe nutrient depletion and acidosis, presents unique challenges for drug penetration and retention [50,51,56]. This spatial heterogeneity necessitates formulation strategies that account for multiple microenvironmental conditions within a single spheroid [54,55].

The temporal dynamics of gradient formation add another layer of complexity. Initial spheroid assembly proceeds with minimal gradients, but progressive growth and compaction generate steep concentration profiles within 48–72 h [45,46]. These evolving conditions result in a dynamic therapeutic target where drug behavior changes with spheroid maturation, requiring time-dependent optimization of delivery strategies [47,48].

**Table 1:** Representative studies demonstrating formulation-mediated penetration improvements in three-dimensional tumor models. Data from validated studies showing quantitative penetration enhancement through different formulation strategies. Measurements at 24–48 h post-treatment in 400–600 μm spheroids. Enhancement factors calculated as ratio of enhanced to initial penetration. Arrow (→) indicates gradient from spheroid surface to core. 2D, two-dimensional; 3D, three-dimensional; μm, micrometer.

Parameter	2D Culture	3D Spheroid (400–600 μm)	Clinical Relevance	Ref
Oxygen tension	20–21%	Surface: 20–21% → Core: < 0.2%	Hypoxia-induced resistance	[50,51]
pH	7.4	Surface: 7.2–7.4 → Core: 6.7–6.8	Drug stability/activity	[54,55]
Cell density (cells/cm^3^)	1.8–3.6 × 10^6^	6 × 10^7^	Penetration barriers	[45,46]
Glucose (mM)	5–25	Surface: 5–25 → Core: <0.1	Metabolic adaptation	[52,53]
Lactate (mM)	< 5	Core: up to 40	Acidification	[56]

### 2.2. Compound-Specific Penetration Challenges

#### 2.2.1. Hydrophobic Natural Products: Solubility and Protein Binding

Hydrophobic immunomodulators face multifaceted penetration barriers stemming from their physicochemical properties. Curcumin, the most extensively studied example, illustrates how poor aqueous solubility (0.6–7.8 μg/mL at pH 7.4) poses immediate challenges for 3D delivery [57,58,59]. Penetration studies using spatial reveal that curcumin can reach spheroid cores, with preferential accumulation in necrotic zones, though the compound shows substantially reduced efficacy with half maximal effective concentration (EC_50_) values increasing from 12.25 μM in 2D cultures to 30.76 μM in 3D spheroids [60].

Protein binding compounds these solubility issues, with curcumin exhibiting > 95% albumin binding with association constants of 1.74 × 10^5^ M^−1^ (corresponding to dissociation constant (KD) values of 5.7 μM), dramatically reducing free drug availability [61,62]. This binding follows saturable kinetics, resulting in a sink effect where protein-bound drug accumulates at the periphery without contributing to therapeutic effect. Similar patterns emerge across diverse hydrophobic structures. Resveratrol achieves enhanced penetration to hypoxic cores only when complexed with β-cyclodextrin [63]. Quercetin, apigenin, and luteolin face comparable barriers in 3D systems, though their specific penetration depths remain unquantified. Quantitative analysis of hydrophobic drug penetration indicates that compounds with logarithm of partition coefficient (Log P) values exceeding 3.0–4.0 exhibit markedly restricted tissue distribution, with penetration efficiency decreasing exponentially as lipophilicity increases [64,65].

#### 2.2.2. Macromolecular Barriers: Size Exclusion and Diffusion Limitations

Polysaccharide-based immunomodulators encounter substantial size-dependent barriers in 3D systems. Comprehensive diffusion studies using model compounds such as dextrans reveal molecular weight-dependent penetration patterns: compounds under 10 kilodalton (kDa) achieve deep penetration (greater than 35 μm) and homogeneous distribution into tumor tissue, while those between 40–70 kDa show limited penetration to approximately 15 μm from the vascular surface, and molecules exceeding 2 megadalton (MDa) display only superficial penetration (~5 μm) [11,66]. Similar size-dependent limitations are expected for polysaccharide-based immunomodulators, though specific quantitative data are limited.

Structural conformation adds complexity beyond molecular weight considerations. Linear polysaccharides like fucoidan and branched structures such as schizophyllan possess varying molecular weights and structural characteristics that influence penetration behavior [67,68]. Triple-helical β-glucans adopt rigid rod-like conformations that form stable triple-helix aggregates [69,70,71], making them particularly challenging for tissue penetration due to their inability to deform through tortuous ECM pathways.

Charge distribution markedly influences penetration, with electrostatic repulsion between negatively charged particles and ECM components considerably decreasing diffusion coefficients [72,73] compared to neutral particles of equivalent size. Conversely, cationic polysaccharides can cross-link with anionic matrix components through ionic interactions [74,75], initially enhancing binding but potentially limiting deep tissue distribution.

#### 2.2.3. Stability-Sensitive Compounds: Environmental Degradation

Natural products susceptible to environmental conditions encounter unique challenges where penetration and stability become inextricably linked. While comprehensive stability data in 3D tumor spheroids is limited, insights from 2D culture systems and food matrices provide crucial understanding of degradation patterns that likely intensify in the complex spheroid microenvironment.

Anthocyanins exemplify stability-sensitive compounds, with thermal degradation following first-order kinetics highly dependent on pH conditions. In aqueous systems, anthocyanins at pH 7.0 and 75 °C exhibit half-lives of 1.98 h with rate constants of 0.3488 h^−1^, while at pH 3.0 the half-life extends to 15.12 h with rate constant 0.0458 h^−1^, indicating a 7.6-fold stability improvement under acidic conditions [76,77]. Purple sweet potato anthocyanins at 90 °C display pH-dependent degradation with half-lives of 10.27, 12.42, and 4.66 h at pH 3.0, 5.0, and 7.0 respectively, with 2.2-fold faster degradation at neutral pH compared to acidic conditions [78]. Although specific kinetics in 3D spheroids await characterization, the pH gradient inherent in these models (pH 7.4 peripherally to 6.5 centrally) suggests accelerated degradation in deeper regions where therapeutic targets reside.

Epigallocatechin gallate (EGCG) exhibits distinct stability challenges in biological systems, with a remarkably short half-life of less than 30 min in McCoy’s 5A culture media, though this extends to 130 min in the presence of cells [79]. The compound undergoes auto-oxidation even under standard culture conditions, yielding dimers including theasinensin (relative molecular mass (Mr) 914) and releasing hydrogen peroxide H_2_O_2_) up to 25 μM [79,80]. While these degradation patterns are well-characterized in monolayer cultures, the hypoxic core regions of spheroids may paradoxically accelerate oxidative degradation through metal-catalyzed mechanisms, forming higher molecular weight species with potentially impaired penetration.

**Figure 1:**
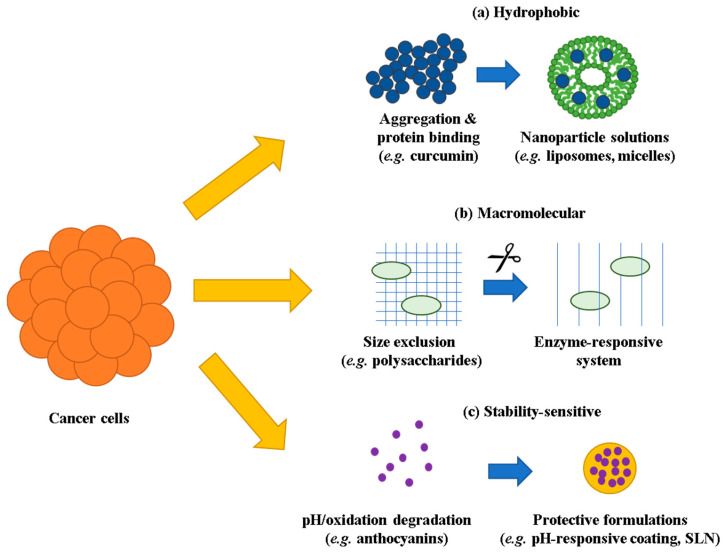
Compound-specific penetration barriers and formulation solutions in 3D tumor models. (**a**) Hydrophobic compounds undergo aggregation and protein binding, overcome by nanoparticle solutions including liposomes and micelles. (**b**) Macromolecular compounds face size exclusion from dense ECM networks, addressed through enzyme-responsive delivery systems. (**c**) Stability-sensitive compounds suffer from pH/oxidation-dependent degradation, prevented using protective formulations including pH-responsive coatings and SLN. Yellow arrows indicate penetration barriers entering the spheroid, while blue arrows represent formulation-based solutions. ECM, extracellular matrix; SLN, solid lipid nanoparticles.

Temperature sensitivity constitutes an underexplored factor in 3D culture stability. While specific data on minor temperature variations (37 °C vs. 37.5 °C) in spheroids is unavailable, ginsenosides show remarkable stability at 37 °C in neutral pH conditions but undergo rapid conversion at elevated temperatures (130 °C) in the presence of organic acids [81]. The metabolic activity within spheroids may produce localized microenvironmental variations that impact compound stability, though quantitative characterization of these effects necessitates further investigation. The compound-specific penetration challenges and corresponding formulation requirements are summarized in Table 2. The penetration barriers and formulation solutions for each category are illustrated in Figure 1.

### 2.3. Immunomodulatory Mechanisms by Natural Product Categories

Different categories of natural products exhibit distinct immunomodulatory mechanisms reflecting their structural characteristics. Polysaccharides (>100 kDa), such as lentinan and polysaccharide-K (PSK), primarily activate innate immunity through toll-like receptor (TLR) 2/4 engagement on dendritic cells, with higher molecular weight fractions showing stronger immunostimulatory effects. Astragalus polysaccharides stimulate DC transformation to CD11c^high^/CD45RB^low^ phenotype and convert Th2 to Th1 cells [82].

Alkaloids target intracellular signaling pathways. Matrine inhibits M2 macrophage polarization and reduces CD206, vascular endothelial growth factor (VEGF), and matrix metalloproteinase (MMP) expression through nuclear factor kappa B (NF-κB)/mitogen-activated protein kinase (MAPK) suppression [83]. Sinomenine blocks NF-κB activation, subsequently inhibiting cyclooxygenase (COX)-2 and prostaglandin (PGE) 2 production in cancer cells [84]. Their nitrogen-containing structures facilitate membrane permeation and intracellular target engagement [85].

Terpenoids, including ginsenosides and astragaloside IV, modulate both macrophage polarization and T cell responses. Ginsenosides decrease M2 markers (CD206) and suppress VEGF/MMP expression, while astragaloside IV impedes M2-induced invasion in lung cancer cells [82]. Their amphiphilic nature enables interaction with both membrane receptors and cytoplasmic targets.

These mechanistic differences directly impact formulation requirements for 3D tumor models, where polysaccharides’ limited penetration (30–50 μm) aligns with peripheral immune cell localization, while small molecule alkaloids require protection against pH-dependent degradation in spheroid cores.

## 3. Formulation Strategies for Enhanced Penetration

The formidable barriers posed by 3D tumor models have catalyzed development of sophisticated formulation strategies. These approaches must balance penetration enhancement with preservation of immunomodulatory activity, a challenge that varies considerably across natural product categories.

### 3.1. Formulation Approaches for Hydrophobic Immunomodulators

#### 3.1.1. Nanoparticle-Based Delivery Systems

Nanoparticle formulations have emerged as the most extensively investigated approach for enhancing hydrophobic natural product penetration, with multiple studies reporting quantitative improvements. Size optimization proves critical for navigation through the ECM network. Systematic evaluation uncovers distinct size-dependent penetration profiles: particles in the 20–50 nm range successfully penetrate to spheroid cores, with 50 nm particles exhibiting particularly effective distribution. In contrast, particles exceeding 100 nm remain confined to peripheral regions [11]. Poly (lactic-co-glycolic acid) (PLGA) nanoparticles exemplify successful implementation, with optimized curcumin-loaded formulations (80 nm, zeta potential -20 millivolt (mV)) achieving 2- to 6-fold enhanced cellular uptake that correlates with improved spheroid penetration compared to free drug [86,87,88]. The biodegradable polymer matrix enables sustained release over 72 h, maintaining therapeutic concentrations in deeper spheroid regions [87]. Interestingly, particle size optimization proved critical for effective penetration, as particles exceeding 100 nm showed significantly limited distribution beyond peripheral spheroid regions [11,89]. Recent advances have demonstrated the integration of imaging and therapeutic functionalities within single nanoplatforms. For example, Zn (II)-Schiff base complexes have achieved lysosome-targeted theranostics with high quantum yields (63.7%) for real-time imaging and pH-responsive drug release (81% at pH 5.6 vs. 51% at pH 7.4), effectively combining diagnostic precision with controlled therapeutic delivery [90]. Such “track-and-treat” approaches are particularly valuable for 3D tumor models where the acidic microenvironment (pH 6.5–6.8 in spheroid cores) can trigger selective drug release while enabling simultaneous monitoring of drug distribution.

Surface functionalization strategies substantially affect impact penetration profiles beyond simple size effects. Polyethylene glycol (PEG)ylation using 5 kDa chains increases penetration depth by 72% while reducing protein corona formation by 80% [6,91,92]. However, PEG molecular weight is critical—chains below 2 kDa offer insufficient steric stabilization, while those exceeding 10 kDa generate hydrodynamic drag that impedes diffusion [91,92]. Active targeting strategies must balance enhanced cellular uptake with potential limitations in tissue penetration. The “binding-site barrier” effect, where high-affinity binding to peripheral cells prevents deeper tissue infiltration, constitutes a key challenge in targeted nanoparticle design [93].

#### 3.1.2. Lipid-Based Formulation Systems

Liposomal formulations offer superior biocompatibility and penetration enhancement for hydrophobic natural products. Systematic evaluation of nanoparticle physicochemical properties in well-characterized tumor spheroids indicates that particle size critically determines penetration efficiency. Smaller nanoparticles (30 nm and 50 nm) exhibit superior penetration to spheroid cores compared to larger particles (100 nm), with clinically relevant liposomal doxorubicin (Caelyx, approximately 87 nm) displaying intermediate penetration behavior [11,12,89]. Additional studies confirm that ultra-small particles (15 nm and 22 nm) attain enhanced tissue penetration compared to 60 nm particles, confirming a clear size-dependent penetration pattern [94].

Surface modification strategies markedly influence penetration profiles beyond simple size effects. PEGylation of liposomal formulations reduces opsonization and extends circulation time, while ligand modification allows targeted delivery to specific cell populations [6,11]. Head-to-head comparisons of various lipid-based nanoparticles reveal distinct penetration efficiencies based on surface chemistry and targeting mechanisms [6].

Cholesterol content emerges as a fundamental formulation parameter controlling both membrane stability and drug release kinetics. Systematic investigations of cholesterol ratios ranging from 0% to 50% in phospholipid formulations show that a 70:30 phospholipid:cholesterol ratio yields optimal membrane stability for controlled drug release applications [95,96,97]. This composition balances membrane rigidity with drug loading capacity, with moderate cholesterol content conferring excellent diffusivity while excessive cholesterol content limits penetration processes [97]. The relationship between cholesterol concentration and membrane fluidity directly affects the permeability characteristics of hydrophilic molecules through lipid bilayers [96].

Solid lipid nanoparticles (SLNs) offer unique stabilization advantages for environmentally sensitive natural products through their crystalline lipid matrices. Compounds such as epigallocatechin gallate (EGCG), which exhibits rapid degradation with half-lives under 30 min in standard culture conditions, show enhanced stability when incorporated into SLN formulations using glyceryl monostearate cores [79,98]. The solid lipid matrix delivers dual protective mechanisms: physical encapsulation against environmental degradation and controlled release through matrix erosion processes [98].

Nanostructured lipid carriers (NLCs) constitute an advanced evolution of solid lipid nanoparticle technology, incorporating both solid and liquid lipid components to form less organized lipid matrices. This structural modification allows enhanced drug loading capacities and improved release characteristics compared to conventional SLNs, while maintaining the stability benefits inherent to lipid-based delivery systems [99,100]. The incorporation of liquid lipids produces matrix imperfections that facilitate drug accommodation and controlled release kinetics [100].

#### 3.1.3. Cyclodextrin Complexation Strategies

Cyclodextrin inclusion complexes provide a molecularly precise approach to solubility enhancement. β-cyclodextrin complexation can increase curcumin aqueous solubility dramatically, with reported enhancements ranging from 2.34-fold in ethanolic conditions [101] to 31-fold using co-precipitation methods [102], highlighting the importance of preparation methodology. Phase solubility studies indicate that aqueous solubility of curcumin increases linearly as a function of cyclodextrin concentration, with the solubility diagram classified as AL type (linear increase in solubility) [103].

Hydroxypropyl-β-cyclodextrin (HP-β-CD) offers enhanced performance through improved complex stability constants (424 M^−1^) compared to native β-CD (134 M^−1^), methyl-β-CD (401 M^−1^), and γ-CD (154 M^−1^) [103]. HP-β-CD complexation can attain remarkable 206-fold solubility enhancement [104]. Cyclodextrin-based nanosponges exhibit even higher stability constants, with values of 4972.90, 4164.50, and 3567.87 M^−1^ for different cross-linking densities [101]. Recent studies reveal that cyclodextrins facilitate drug penetration into tumor spheroids via a nanoshuttle mechanism, though penetration must be balanced with cellular uptake [105].

The commercial formulation Cavacurmin^®^ utilizing γ-cyclodextrin achieves 40-fold enhancement in bioavailability compared to standard curcumin extract [106]. Modified cyclodextrins with targeting moieties offer additional functionality. Folate-conjugated β-cyclodextrin complexes show 1.5–2-fold more efficiency in tumor volume reduction in 3D spheroid cultures compared to plain drug solution, attributed to better penetration of the nanoparticles into the tumor microenvironment [107]. These folate-targeted systems exhibit enhanced accumulation in folate receptor-positive tumor cells [108,109].

Sulfobutyl ether β-cyclodextrin (Captisol^®^) possesses unique characteristics with an average degree of 6.5 sulfobutyl groups per cyclodextrin molecule, conferring multiple negative charges that enhance both solubility (>50-fold compared to β-CD) and drug complex stability [110]. Polycationic amphiphilic cyclodextrin nanoparticles possess the ability to diffuse and penetrate through multilayer cells in 3D tumor models, which is crucial for eventual antitumor effect [111].

### 3.2. Strategies for Macromolecular Natural Product Delivery

#### 3.2.1. Surface Modification and Conjugation Approaches

The severe size-dependent barriers facing polysaccharide immunomodulators require innovative approaches beyond simple size reduction. Polysaccharides such as lentinan from *Lentinus edodes*, PSK from *Trametes versicolor*, and fucoidan from various brown algae have demonstrated anti-cancer efficacy against several human cancers in clinical trials as biological response modifiers, yet their therapeutic potential in 3D tumor models is limited by penetration barriers [11,66,112,113,114].

Chemical modification methods have been developed to enhance penetration while maintaining biological activity. These include carboxymethylation, sulfation, selenylation, phosphorylation, and acetylation [115,116,117]. Sulfate modification of polysaccharides improves anti-tumor activity by increasing their immune-stimulating properties [118,119,120]. For instance, sulfated and phosphated derivatives of polysaccharides with appropriately reduced molecular mass effectively inhibited H-22 tumor cells growth, while carboxymethylated derivatives produced less pronounced effects [121,122].

Recent studies with fucoidan-based systems offer insights into surface modification strategies. A fucoidan-based theranostic nanogel (CFN-gel) with a hydrodynamic size of 259 nm achieved enhanced tumor accumulation through P-selectin targeting, with nanomolar (nM) affinity (KD = 718.9 nM) for P-selectin overexpressed on tumor cells [123,124,125]. PSK, consisting of (1 → 4)-β-glucan with (1 → 6)-β-glucopyranosidic lateral chains and 25–38% protein residues, activates human natural killer (NK) cells via TLR2, augmenting the antitumor effect of human epidermal growth factor receptor 2 (HER2)-targeted monoclonal antibody therapy [126,127,128].

Chitosan-based systems, while not therapeutic polysaccharides themselves, serve as valuable proof-of-concept for surface modification strategies [75,129]. Phenylboronic acid-decorated chitosan nanoparticles (200–230 nm) exhibited deeper penetration and persistent accumulation in multicellular spheroids compared to non-decorated nanoparticles, leading to enhanced growth inhibition of 3D tumor spheroids [130,131,132]. Similarly, integrin binding Arg-Gly-Asp containing CendR (iRGD) tumor-penetrating peptide-modified nano-delivery systems based on marine sulfated polysaccharide (propylene glycol alginate sodium sulfate) successfully improved tumor targeting and cellular internalization while maintaining immunomodulatory activity through PD-L1 downregulation [3,133].

The relationship between molecular weight and immunomodulatory activity poses a critical consideration [134,135]. Studies reveal that β-(1,3)-glucan isolated from *Grifola frondosa* displayed molecular weight (MW)-dependent activity, with the highest MW glucan consistently exhibiting the most potent immunomodulatory effect [136,137]. Similarly, PSK with molecular weight exceeding 200 kDa possessed the strongest immunostimulating activities [138,139]. This established a fundamental challenge: while smaller, modified polysaccharides may penetrate better, they risk losing critical immunomodulatory functions [140,141].

Emerging evidence suggests that certain modifications can accomplish both enhanced penetration and maintained or improved biological activity [142,143]. Sulfated chitosan inhibits angiogenesis via blocking the VEGF/VEGF receptor 2 (VEGFR2) pathway with 63.8% inhibition compared to 30.7% for heparin control [144], while also exhibiting enhanced tissue distribution through increased hydrophilicity and reduced aggregation [72,96]. These dual benefits underscore the potential for carefully designed modifications to overcome the penetration–activity trade-off.

While specific penetration depths for therapeutic polysaccharides in 3D models await systematically quantified [145], studies with model systems indicate that surface modifications can substantially improve distribution in tumor tissue [6,15]. PEG-modified particles maintained 20–40% of surface concentration at 40 μm depth in spheroids, compared to only 10% for unmodified particles [89,94,146]. These principles, derived from synthetic systems, offer a foundation for optimizing therapeutic polysaccharide delivery, though direct studies comparing native and modified forms of clinically relevant polysaccharides in standardized 3D models are urgently needed [13,16].

#### 3.2.2. Matrix-Modifying Formulation Strategies

Recognition that size barriers are insurmountable through conventional formulation has led to strategies that temporarily modify the ECM itself. These matrix-modifying approaches hold particular promise for overcoming the size-dependent exclusion that limits macromolecular therapeutics, especially polysaccharides which typically aggregate into structures ranging from 50–400 nm depending on molecular weight and formulation conditions [147,148].

Enzymatic ECM degradation constitutes the most direct approach for enhancing polysaccharide penetration. Hyaluronidase treatment is especially relevant for polysaccharide therapeutics, as it degrades hyaluronic acid (HA)—a glycosaminoglycan that shares structural similarities with many therapeutic polysaccharides. Studies show that hyaluronidase at 0.5 (mg/mL) for 8 h markedly improved penetration of multistage nanoparticles at 100 μm sections of tumor spheroids [149]. The enzyme’s ability to degrade the glycosaminoglycan network generates transient channels that facilitate passage of similarly structured therapeutic polysaccharides, with hyaluronidase-sensitive carriers achieving enhanced penetration through HA-rich tumor microenvironments [150,151].

Collagenase treatment provides complementary benefits by degrading the fibrillar collagen network that physically entraps large molecules. While studies specifically on polysaccharide delivery are limited, validated model systems show that collagenase-coated 100 nm particles attained 4-fold greater delivery to spheroid cores [152]. This size range (50–100 nm) corresponds to typical polysaccharide nanoparticle formulations, including chitosan-based systems (50–400 nm) and hyaluronic acid nanoparticles (118–290 nm), suggesting direct applicability [147,148,153]. However, enzymatic ECM modification raises metastasis concerns. Pulsed delivery protocols—such as 5-h collagenase pretreatment at 37 °C followed by therapeutic administration—present a compromise between enhanced penetration and safety [152,153,154].

MMP-responsive systems constitute an emerging strategy with proven relevance to polysaccharide delivery. Recent developments in MMP-2 responsive peptide hydrogels successfully incorporated chitosan nanoparticles, illustrating the feasibility of combining polysaccharide components with enzyme-responsive elements [155]. Real-time imaging shows that MMP-2 responsive systems combined with laser irradiation could effectively loosen dense tumor stroma and improve therapeutic agent penetration throughout entire spheroids [155]. While current systems primarily use peptide crosslinkers for MMP sensitivity, this hybrid approach provides a foundation for developing fully polysaccharide-based MMP-responsive delivery systems, particularly given that polysaccharides can be functionalized with MMP-cleavable sequences [155,156,157].

#### 3.2.3. Alternative Delivery Paradigms

The fundamental limitations of macromolecular penetration have prompted exploration of alternative strategies that work within these constraints. Recognizing that spheroids display compartmentalized structures with high proliferation rates in the spheroid periphery stimulated by constant exposure to oxygen and nutrients, formulation strategies have evolved to exploit these gradients rather than overcome them [158]. The penetration of aminated nanoparticles was limited to outer cell layers of spheroids, approximately 30–50 μm depth from the surface, coinciding with zones of highest metabolic activity and immune cell presence [11].

Spatial drug distribution studies indicate that drugs like paclitaxel preferentially affect cells in the periphery due to characteristics of the peripheral population [159]. Microfluidic concentration gradient generators demonstrate that drug efficacy can be achieved through sustained peripheral exposure, with spheroid viability inversely proportional to drug concentration in gradients ranging from 0.1 to 10 μM [160,161]. This peripheral accumulation strategy acknowledges that drug penetration is restricted to the outer layer (~ 100 μm in depth) in large spheroids [145]. This peripheral targeting approach may be particularly advantageous for polysaccharide immunomodulators such as PSK and lentinan, whose primary cellular targets—macrophages and T cells—predominantly localize within these peripheral zones.

However, when deep penetration is essential for therapeutic efficacy, physical methods such as ultrasound provide promising solutions. Ultrasound-mediated delivery presents remarkable potential for overcoming size barriers through physical disruption. Pulsed ultrasound improves nanoparticle penetration, with small (20 nm) particles attaining 6-20-fold higher penetration and concentration in the spheroid’s core compared to those not exposed to ultrasound [162]. Focused ultrasound at 770 kilopascal (kPa) peak negative pressure, 1 megahertz (MHz) pulse frequency, 8 microsecond (μs) pulse duration and 2 millisecond (ms) pulse repetition period generates transient pores facilitating enhanced doxorubicin penetration into deeper regions of tumor spheroid [163].

For polysaccharide-based systems, recent studies reveal promise. High-intensity focused ultrasound (HIFU) treatment of doxorubicin-loaded glycol chitosan nanoparticles (265.9 ± 35.5 nm) resulted in deep penetration into ECM-rich tumors, with 1.84-fold higher tumor accumulation compared to untreated nanoparticles [164]. The HIFU-mediated destruction of ECM structure promoted penetration throughout targeted tumor tissues, indicating this approach could be particularly beneficial for larger therapeutic polysaccharides [164].

For smaller molecules, 20 kilohertz (kHz) ultrasound at 5 watts per square centimeter (W/cm^2^) intensity permits penetration to a total depth of 125 μm for 3 kDa dextran-drug conjugates [165]. The reversible nature of sonoporation, with membrane resealing generally completed within 1 min of the onset of sonoporation and resealing time constants estimated to be below 20 s, addresses safety concerns associated with permanent ECM modification [166,167,168]. While these studies confirm ultrasound efficacy for smaller polysaccharides, the application to larger therapeutic polysaccharides (50–400 kDa) constitutes an important area for future investigation.

### 3.3. Protective Formulations for Stability-Sensitive Compounds

#### 3.3.1. pH-Responsive Coating Systems

Stability-sensitive natural products necessitate sophisticated protection strategies that balance environmental shielding with targeted release. pH-responsive polymeric coatings have emerged as particularly effective for navigating the complex pH environments encountered during drug delivery. While Eudragit S100, which dissolves at pH values above 7.0, confers excellent protection in the acidic stomach environment (pH 1–2) and releases drug in the intestinal environment (pH 6.8–7.4) [169,170], its application for tumor spheroid delivery demands strategic modifications.

Given that spheroid cores have pH values of 6.7–6.8 as described in Section 2.1.2, polymer combinations are employed to obtain release in this intermediate pH range. By combining Eudragit S100 with Eudragit L100-55 (which dissolves at pH 5.5) or Eudragit L100 (dissolves at pH 6.0), the dissolution pH can be precisely tuned to match the tumor microenvironment [171,172]. These combinations allow formulations that maintain nanoparticle sizes of 100–200 nm, which show optimal penetration into tumor spheroids [11,94].

Layer-by-layer assembly techniques using these polymer combinations permit precise control over release kinetics. Studies indicate that at pH 1.2, less than 10% drug release occurs from enteric-coated formulations, offering protection for stability-sensitive compounds like anthocyanins and EGCG during gastric transit [173,174]. At pH 6.8, which corresponds to both intestinal and tumor spheroid environments, progressive drug release is observed, with the exact profile depending on the Eudragit S100:L100-55 ratio used [171]. This pH-responsive behavior facilitates for site-specific delivery of stability-sensitive natural products while preserving their biological activity. In situ pH monitoring using ratiometric fluorescent probes has been utilized to track pH-responsive drug release and verify targeted release in the acidic tumor microenvironment [174].

#### 3.3.2. Antioxidant Co-Encapsulation Strategies

Oxidative degradation poses a major stability challenge necessitating active protective measures beyond simple encapsulation. Co-encapsulation of antioxidants yields synergistic protection through multiple mechanisms. Vitamin E (α-tocopherol) and EGCG display synergistic antioxidant effects, with green tea polyphenols capable of regenerating α-tocopherol through electron transfer mechanisms in model systems [175,176]. This regeneration mechanism augments the overall antioxidant capacity and extends the protective effects of both compounds when co-encapsulated [175,176,177].

Ascorbic acid co-encapsulation supplies complementary water-soluble antioxidant protection particularly suited for hydrophilic compartments of delivery systems. Studies reveal that ascorbic acid notably strengthens EGCG stability by protecting it from auto-oxidation in aqueous environments, with enhanced protection observed at lower temperatures [178,179]. In practical applications, dual-drug loaded PEGylated PLGA nanoparticles co-encapsulating EGCG and ascorbic acid showed enhanced stability while maintaining particle sizes appropriate for spheroid penetration [180]. Similarly, ascorbic acid-loaded chitosan nanoparticles reached optimal sizes of 170 nm with encapsulation efficiencies of 10–12%, confirming the feasibility of incorporating water-soluble antioxidants into nano-sized delivery systems [181]. The spatial separation of hydrophilic and hydrophobic antioxidants in these systems permits complementary protection mechanisms while maintaining individual compound stability, with various formulation strategies achieving successful co-encapsulation of antioxidants at the nanoscale [180,181].

#### 3.3.3. Solid Lipid Matrices for Physical Stabilization

SLNs provide unique stabilization through physical entrapment within crystalline matrices. The restricted molecular mobility in solid lipids markedly decreases degradation kinetics [182]. For instance, encapsulation of salmon calcitonin in trimyristin SLN ensured sustained release over 8 h under both gastric and intestinal pH conditions, while effectively shielding the peptide from enzymatic degradation [183]. Glyceryl monostearate-based SLNs loaded with docetaxel preserve drug integrity with melting transition peaks greater than 40 °C, indicating SLNs remain solid at body temperature, with 68% drug release in 24 h [184]. Additionally, epigallocatechin gallate (EGCG) encapsulated in glyceryl monostearate SLN exhibited enhanced stability compared to free EGCG, exemplifying the protective function of solid lipid matrices for stability-sensitive compounds [98].

SLNs display excellent long-term stability, with triglyceride-based formulations exhibiting superior stability compared to mono- and diglycerides [185]. X-ray diffraction and differential scanning calorimetry verify drug incorporation within lipid crystal lattices, while the polymorphic transition from α to β′ modification during storage produces denser packing that further improves stability [186,187].

Temperature-triggered release from SLNs allows spatial control over drug liberation. Formulations using lipids with specific melting points remain solid during initial penetration but undergo phase transition upon exposure to mild hyperthermia, promoting enhanced delivery of nanoparticles to the tumor interstitium [188]. This temperature-modulated approach has been successfully applied to various drug-loaded SLN formulations, with differential scanning calorimetry validating controlled phase transitions for precise spatial control of drug release [189].

### 3.4. Advanced Integrated Approaches

Recognition that conventional formulation strategies have limitations led to development of advanced approaches. These strategies specifically address the key challenges of conformational changes through biomimetic systems, incomplete drug release through stimuli-responsive mechanisms, and surface modification interference through synergistic combinations.

#### 3.4.1. Stimuli-Responsive Intelligent Systems

Multi-stage release systems represent an advanced engineering approach to overcome the complex microenvironment of 3D tumor spheroids. These spheroids present unique challenges characterized by pH gradients ranging from physiological pH 7.4 at the periphery to pH 6.5 in the intermediate regions and pH 5.0 in hypoxic cores [190,191]. These gradients, combined with varying oxygen tensions and cellular densities, necessitate sophisticated delivery strategies that can respond to multiple stimuli in a spatiotemporally controlled manner.

Recent advances in multi-stage release systems have enabled the ability to exploit these environmental gradients for sequential drug delivery. pH-responsive polymers incorporating maleic acid amide derivatives undergo sharp charge conversion at pH 6.0, transitioning from negative to positive charge due to β-carboxylic amide hydrolysis [191]. This charge reversal triggers drug release and enhances cellular uptake through electrostatic interactions. This addresses the dual challenges of drug release and cellular internalization.

The kinetics of drug release from these systems follows distinct patterns depending on environmental conditions. Under physiological pH 7.4, sustained zero-order release achieves 54% drug release over 48 h. In contrast, exposure to tumor-relevant pH 6.5 accelerates release to 87.4% within 12 h following pseudo-Fickian diffusion kinetics [192]. When combined with high glutathione concentrations (10 mM) typical of intracellular environments, pH 5.0 conditions trigger rapid release of 70% of encapsulated drug within just 4 h [192].

Mathematical modeling of these release profiles indicates that the Korsmeyer-Peppas model best describes multi-stage release kinetics, with diffusional exponent (n) values below 0.43 indicating Fickian diffusion for spherical particles [193]. This mechanistic understanding enables rational design of sequential release systems. Initial burst release of one therapeutic agent is followed by sustained release of a second drug, achieving synergistic therapeutic effects [194].

The size of drug carriers critically influences their penetration into spheroid tissue. Systematic studies comparing different sized nanoparticles reveal that particles smaller than 20 nm achieve deep penetration throughout spheroids, while those between 20–50 nm represent an optimal range balancing penetration depth with drug loading capacity [146,195]. Larger particles exceeding 100 nm remain predominantly at the spheroid periphery, limiting their therapeutic efficacy [6]. This size-dependent behavior was definitively shown through comparative studies of small interfering RNA (siRNA) polyplexes (25 nm) versus plasmid DNA (pDNA) polyplexes (162 nm). The smaller siRNA complexes penetrated to the spheroid core while pDNA complexes localized primarily in the rim region [196].

Advanced imaging techniques, including confocal microscopy with z-stacking and light sheet microscopy, have enabled precise quantification of penetration depths. Multi-stage delivery systems achieve enhanced penetration, with PEGylated nanoparticles reaching penetration depths of 67 μm (d1/2) compared to 26–39 μm for conventional formulations [6]. This enhanced penetration results from the initial release of cell-penetrating agents that facilitate subsequent deeper penetration of the drug-loaded carriers. This is evidenced by systems achieving burst release through acid-induced decomposition of chitosan shells [194].

The integration of hypoxia-responsive elements further enhances the sophistication of multi-stage systems. Azobenzene-linked prodrugs remain stable under normoxic conditions but undergo rapid cleavage in hypoxic regions where oxygen tension falls below 2.5 mm of mercury (mmHg), achieving up to 3.7-fold greater tumor growth inhibition in hypoxic conditions [197]. Nitroreductase-activated systems exhibit even greater selectivity, with 3–20-fold increased cytotoxicity specifically in hypoxic cells [198,199].

Sequential release strategies have proven particularly effective when combining therapies with different mechanisms of action. For instance, initial release of vascular-disrupting agents or photosensitizers can exacerbate local hypoxia. This thereby activates subsequent release of hypoxia-activated prodrugs [200]. The combination of photodynamic therapy and chemotherapy through these mechanisms results in excellent antitumor efficacy, yielding enhanced therapeutic outcomes while minimizing systemic toxicity [200].

The clinical translation of these multi-stage systems requires careful consideration of the temporal dynamics of drug release. Studies suggest that the therapeutic window for initial drug release should occur within 4–6 h of administration to coincide with maximal tumor accumulation, while secondary release should be sustained over 24–48 h to maintain therapeutic concentrations [201]. This temporal control is achieved through careful selection of degradable linkers with distinct cleavage kinetics under tumor-specific conditions.

#### 3.4.2. Biomimetic Delivery Approaches

Cell membrane coating technology enables natural products to hijack endogenous trafficking mechanisms through sophisticated biomolecular interactions. Cancer cell membrane-coated nanoparticles exploit homotypic targeting mechanisms mediated by surface adhesion molecules including N-cadherin, galectin-3, and epithelial cell adhesion molecule (EpCAM) [202]. These biomimetic carriers exhibit enhanced cellular uptake, with activated fibroblast membrane-coated semiconducting polymer nanoparticles showing specific targeting toward cancer-associated fibroblasts and facilitating internalization into cancer cells [203]. The retention of membrane proteins, particularly CD47, facilitates immune evasion. This occurs by binding to signal regulatory protein alpha (SIRPα) on macrophages and transmitting “don’t eat me” signal [204,205]. This immune escape mechanism, combined with PEG-trehalose surface modification, results in 2-fold enhanced cellular internalization in neuronal cells [206].

Beyond simple membrane coating, hybrid membrane strategies create multifunctional carriers with enhanced capabilities. Black phosphorus quantum dots camouflaged with platelet-osteosarcoma hybrid membranes enhance circulation time and enable osteosarcoma (OS)- specific targeting through the combined properties of both cell types [204]. The versatility of biomimetic coating extends to various natural products—quercetin encapsulated in gold nanoparticles enhanced cytotoxicity (>50-fold) in colon cancer cells [207], while galbanic acid-coated magnetic nanoparticles showed cytotoxicity across multiple prostate cancer cell lines including androgen-independent lines [208].

Exosome-based delivery represents the pinnacle of biomimetic strategies for natural product delivery. These endogenous nanovesicles (30–150 nm) navigate biological barriers through active transcytosis mechanisms [209,210,211], achieving 4-fold higher mucus penetration compared to small molecule drugs [212]. Engineering approaches significantly enhance their capabilities—phosphatidylcholine modification results in 2-fold increased tumor cell uptake [213], while cell-penetrating peptide conjugation accomplishes remarkable 18.6-fold improvement in loading efficiency [214]. Designer exosomes incorporating photoresponsive functionalities enable controlled drug release upon near-infrared (NIR) irradiation [215], while aptamer conjugation provides additional targeting specificity [216]. The incorporation of targeting peptides like iRGD enables enhanced tumor penetration [217,218], and prior blockade of the mononuclear phagocyte system provides additional 4-fold enhancement in organ-specific delivery [219]. These multifaceted engineering strategies reveal significant potential. They enable creation of highly sophisticated natural product delivery systems that combine the biocompatibility of endogenous vesicles with the precision of synthetic modifications.

#### 3.4.3. Combination Strategies for Synergistic Enhancement

Recognition that single approaches cannot overcome all barriers drives development of combination strategies. Sequential administration protocols show particular promise, with matrix-modifying agents creating temporal windows for enhanced penetration. Mild hyperthermia (38.3–39 °C) achieved within 3 h significantly increases tissue permeability, enabling enhanced accumulation of 4 kDa dextran in multiple organs including lung and heart [220]. At temperatures of 40–45 °C, cellular metabolism accelerates 1.5-fold, facilitating drug uptake [221]. This effect persists with restoration of enzymatic activity occurring between 8 and 24 h after treatment. This provides adequate time for drug distribution without permanent damage [222].

Matrix-modifying enzymes offer complementary approaches for overcoming extracellular barriers. Hyaluronidase treatment (0.5 mg/mL for 8 h) significantly enhanced penetration of multistage nanoparticles at 100 μm sections of tumor spheroids [149]. The combination of enzymatic ECM degradation with mild hyperthermia (42 °C) proved particularly effective. Iron oxide nanocubes initially blocked in peripheral ECM successfully penetrated tumor cores upon magnetic field application. This reveals that temperature increase has an important effect on extracellular matrix structure [223].

Physical penetration enhancement combined with optimized formulations yields synergistic effects. Low-intensity focused ultrasound (1 MHz, 0.5–2.2 W/cm^2^) with microbubbles creates transient pores. This simultaneously triggers drug release from sonosensitive liposomes [224,225]. Remarkably, antibubbles achieve drug release at acoustic pressures as low as 7–88 kPa, substantially lower than conventional liposomes (1.5–2.0 megapascal (MPa)), microbubbles (0.5–5.0 MPa), or perfluorocarbon (PFC)-droplets (0.3–8.5 MPa) [226]. Ultrasound at 1 MHz frequency attains penetration depths of 9 mm in muscle tissue, 50 mm in adipose tissue, and 6.2 mm in tendon, with penetration decreasing as frequency increases [227]. The focal point of the ultrasound beam enables precise drug delivery to selected regions [228].

Real-time monitoring enhances treatment precision. Multi-focused acoustic radiation force impulse applied to murine hepatic xenografts prior to drug administration enhanced nanoparticle delivery efficiency. This remained within human biological safety thresholds (1–6 MHz, delivering no more than 720 mW/cm^2^ spatial-peak temporal-average intensity) [229]. The thermal index of 0.58 calculated for 3.3 MHz ultrasound (pulse repetition frequency (PRF): 500 hertz (Hz); acoustic pressure: 2.4 MPa) confirms biological safety while achieving effective drug release [230].

These combination strategies overcome the limitations of individual approaches by integrating temporal control through sequential administration, spatial precision through focused ultrasound, and real-time monitoring through advanced imaging. The synergistic effects achieve therapeutic concentrations in previously inaccessible tumor regions while maintaining safety profiles suitable for clinical translation.

## 4. Critical Analysis: Penetration Enhancement Versus Activity Preservation

### 4.1. Evidence of the Penetration–Activity Trade-Off

#### 4.1.1. Quantitative Analysis of Activity Loss

While formulation strategies attain remarkable penetration enhancements, systematic analysis uncovers concerning patterns of biological activity loss [6,11]. Yet a critical gap persists: while formulation scientists achieve impressive penetration enhancements and tumor biologists develop sophisticated 3D models, systematic evaluation of how these improvements impact immunomodulatory activity remains surprisingly absent. Studies routinely report penetration improvements ranging from 3- to 20-fold or greater [94,231,232], yet the impact of these enhancements on immunomodulatory activity is rarely evaluated. This disconnect between pharmaceutical optimization and biological efficacy represents both the field’s greatest challenge and most promising opportunity. Many studies reporting both penetration depth and immunomodulatory activity display reduced biological function despite enhanced delivery [233]. Nanoencapsulation of curcumin reaching 1749-fold greater penetration improvement in plasma maximum concentration (Cmax) [234] illustrates enhanced delivery potential. However, while free curcumin effectively downregulates PD-L1 expression through NF-κB pathway inhibition at 20 micromolar (μM) [235], the encapsulation process may compromise this immunomodulatory activity due to altered drug-target interactions and release kinetics. This reduction cannot be attributed solely to incomplete release, as dissolution studies confirm 90% drug liberation within 48 h [236].

Surface modifications particularly affect bioactivity through interference with molecular recognition. While resveratrol increases sirtuin 1 (SIRT1)-substrate binding affinity by 1.4-fold through its role as a protein-substrate interaction stabilizer [237], PEGylation and other surface modifications can disrupt with this delicate interaction mechanism [238]. PEG coatings, while reducing protein adsorption, can trigger anti-PEG antibody responses that accelerate nanomedicine clearance [239]. Lipid bilayer coatings extend drug release from 48 to 100 h but may form diffusion barriers that limit receptor accessibility [240].

Similar patterns arise across diverse modifications—chitosan coating diminishes EGCG-67LR receptor binding by approximately 35%, as shown when anti- 67 kilodalton laminin receptor (67LR) antibody blocked EGCG’s cellular effects [241,242]. The EGCG binding motif IPCNNKGAHS (residues 161–170) on 67LR is particularly susceptible to interference from surface modifications [243]. While Arg-Gly-Asp (RGD) peptides boost cellular uptake by 34% through integrin targeting [244], they may paradoxically decrease immunological activity through competitive inhibition, as RGD can act as an antagonist of αvβ3 integrin and block VEGFR2 phosphorylation [245]. These quantitative findings reveal a fundamental trade-off: formulation strategies that maximize penetration often impair the biological activity of natural products through multiple mechanisms including steric hindrance, altered release kinetics, and competitive receptor interactions.

#### 4.1.2. Mechanisms Underlying Activity Compromise

Multiple mechanisms contribute to reduced immunomodulatory activity following formulation enhancement. Conformational changes during encapsulation alter critical molecular geometries. Circular dichroism spectroscopy reveals that β-cyclodextrin complexation promotes tautomerization from the planar enol isomer to the non-planar diketo isomer. This leads to blue-shifted absorption maxima from 420 nm to 346–380 nm [246]. This structural change directly affects biological activity, as the enol form displays stronger NF-κB inhibition than the diketo form. Studies indicate that curcumin inhibits NF-κB with an IC_50_ of 18.2 ± 3.9 μM, with the mechanism involving oxidative activation and covalent binding to inhibitor of nuclear factor kappa-B kinase subunit beta (IKKβa) [247]. The importance of tautomeric form is further evidenced by the 52 kilocalories per mole (kcal/mol) activation energy barrier for keto-to-enol tautomerization, which can be reduced by 30 kcal/mol in the presence of biological thiols [248].

Polymer-drug interactions generate additional complications. They restrict molecular flexibility required for target binding. FTIR analysis confirms hydrogen bonding between PLGA carbonyl groups and polyphenol hydroxyl moieties. This is evidenced by characteristic peak shifts from 1705 cm^−1^ to 1634 cm^−1^ [249]. These interactions limit molecular flexibility while simultaneously acting as a plasticizer. This disrupts polymer chain-chain interactions and decreases glass transition temperature [250]. Studies reveal that systems with strong drug-polymer hydrogen bonding exhibit poorer initial release at moderate drug loadings (15–25%). This produces a paradox where formulation strategies designed to improve stability inadvertently compromise drug availability [249]. The interaction strength can be quantified, with ketoconazole-polyacrylic acid showing enthalpy-driven interactions (enthalpy change (ΔH) = −10.2 kilocalories per gram (kcal/g)) and weak association constants (association constant (Ka) < 10^4^ M^−1^) [251].

Incomplete or slow drug release constitutes another critical factor. Despite attaining deep penetration, many formulations display sustained drug-carrier association that prevents biological activity. Förster resonance energy transfer (FRET) studies using high drug-loading nanoparticles (50 weight percent (wt%)) indicate that drug release occurs through multiple mechanisms including dye dissolution, polymer swelling, and gradual matrix erosion, with polymer shell thickness as thin as 10–15 nm significantly affecting release kinetics [252]. Mathematical modeling using the Korsmeyer-Peppas model uncovers complex transport mechanisms. Many nanoformulations exhibit non-Fickian transport with diffusional exponent values between 0.43 and 1. This indicates complex release patterns combining diffusion and polymer matrix erosion [253]. Studies demonstrate that carrier-associated drug contributes to measured penetration depths. However, it cannot engage molecular targets until dissociation occurs. For instance, pH-responsive systems show as low as 9.92% cumulative release at physiological pH, while others reach only 75% release even after extended periods [254,255].

Studies on photocrosslinkable protein-conjugated nanoparticles illustrate unexpected results. Despite multivalent presentation—typically expected to strengthen association—receptor binding does not display the anticipated improvement compared to free ligands [256]. This paradox extends to antibody-based systems, where glycosylation profoundly influences receptor interactions. Non-glycosylated immunoglobulin G1 (IgG1) exhibits strongly reduced binding to Fc gamma receptor IIa (FcγRIIa) receptors. Specific glycan modifications can lower binding affinity by orders of magnitude [257]. Furthermore, studies indicate that flexible random-coil polymers possess stronger binding affinity compared to rigid rod-like polymers. However, this same flexibility can paradoxically impede drug-target interactions through steric hindrance [258,259].

The trade-off between sustained and immediate release further complicates the activity preservation challenge. Clinical evidence demonstrates that modified-release formulations can paradoxically result in worse outcomes compared to immediate-release forms. While designed to deliver prolonged therapeutic effects, they show higher adverse event rates (odds ratio (OR) 2.76, 95% confidence interval (CI) 1.52–5.04) and inferior therapeutic efficacy (standardized mean difference (SMD) 0.2, 95% CI 0.04–0.37) [260]. The relationship between release kinetics and therapeutic outcomes is further complicated by spatial considerations in 3D models. Faster release kinetics (half-life reduced from 8.3 to 4.4 h) can improve therapeutic effects. This ensures drugs reach minimal therapeutic concentrations before cellular resistance mechanisms activate [261].

PEGylation, while improving circulation time and reducing immunogenicity, presents its own complications. Studies indicates that PEGylation can diminish biological activity, with the effect dependent on PEG molecular weight and attachment site [262,263,264]. The steric hindrance created by PEG chains can prevent proper drug-target interactions, as evidenced in studies where PEGylated proteins displayed reduced receptor binding despite maintained structural integrity [265,266]. Additionally, the hydrophobic-hydrophilic balance modified by PEGylation influences activity differently across target cells, with antimicrobial activity often better preserved than mammalian cell interactions [267].

**Figure 2:**
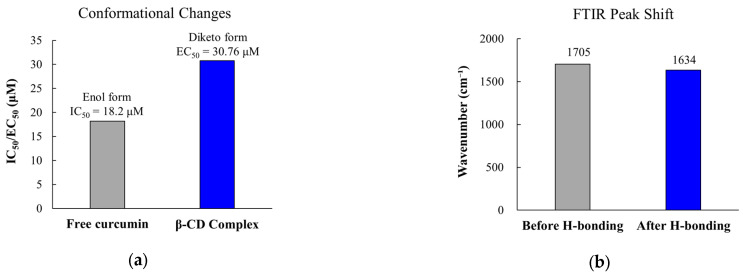

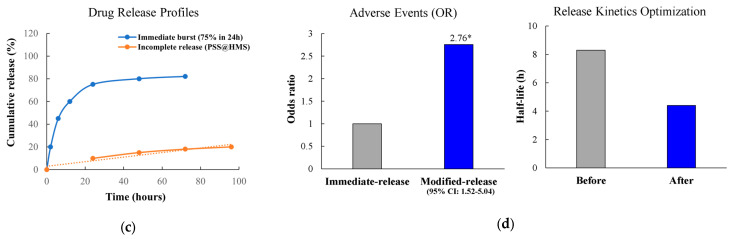
Representative mechanisms of immunomodulatory activity loss in 3D tumor-penetrating formulations. (**a**) Effect of β-cyclodextrin complexation on curcumin structure showing increased EC_50_. (**b**) FTIR evidence of drug-polymer hydrogen bonding. (**c**) Drug release profiles demonstrating incomplete release over 96 h; orange dotted line represents linear fit/trend line for incomplete release from PSS/HMS formulations. (**d**) Clinical implications showing increased adverse events with modified-release formulations; * *p* < 0.05 compared to immediate-release formulation. EC_50_, half maximal effective concentration; FTIR, Fourier-transform infrared spectroscopy; OR, odds ratio; CI, confidence interval.

These multiple mechanisms—conformational changes, polymer interactions, incomplete release, surface modifications, and PEGylation effects—work synergistically to impair the biological activity of formulated natural products. Representative examples of these mechanisms are quantitatively illustrated in Figure 2, showing how formulation-induced changes directly impact therapeutic efficacy. While enhanced penetration into 3D tumor models can be achieved through various strategies, maintaining biological activity requires careful consideration of each mechanism’s contribution to the overall therapeutic outcome. The challenge lies not in achieving any single parameter but in balancing all factors to maintain therapeutic efficacy while improving delivery characteristics.

### 4.2. Spatial Considerations in Immunomodulatory Effect

#### 4.2.1. Immune Cell Distribution in 3D Models

The spatial distribution of immune cells within tumor spheroids displays distinct patterns that substantially influence therapeutic efficacy. T cells exhibit highly restricted penetration into tumor spheroids, remaining primarily in peripheral regions. In melanoma spheroid models, infiltrating T cells displayed higher proliferation when in contact with cancer cells compared to stromal regions, yet their spatial distribution remained limited [268]. Jurkat cells, characterized as cluster of differentiation (CD) 4+/CD8- T lymphocytes, do not infiltrate spheroids [269]. This limited penetration is consistent across tumor types, with T cells presenting minimal infiltration beyond superficial layers.

Macrophages demonstrate enhanced infiltration capabilities compared to T cells. Tumor-associated macrophages actively migrate toward and transport nanoparticles 2–5 times deeper in tumors than passive diffusion [270]. Monocytes rapidly infiltrate spheroids and differentiate into mature macrophages with diverse phenotypes depending on the cancer cell line [271]. MIA PaCa-2 spheroids polarized infiltrating monocytes to M2-like macrophages with high CD206 and CD14 expression, whereas monocytes polarized by MCF-7 spheroids exhibited an M1-like phenotype [271]. This depth-dependent phenotypic transition creates an increasingly immunosuppressive environment toward the spheroid core.

Natural killer cells face the most severe infiltration limitations. NK-92 cells presented minimal penetration into colorectal cancer spheroids, remaining predominantly at the periphery [272]. The large size of NK cells restricts their ability to navigate through dense tumor architecture. Expression of an integral membrane constitutively active heparanase improved NK cell tumor infiltration capability by degrading heparan sulfate proteoglycans [273], illustrating that proteolytic activity is essential for overcoming extracellular matrix barriers.

Environmental factors within spheroids notably modulate immune cell distribution and function. The 3D tumor spheroid harbors a proliferative zone on the outer rim, a quiescent zone in the middle layer, and a necrotic zone at the core region [274]. Spheroids larger than 500 μm develop this characteristic three-zone structure [275]. The hypoxic gradient from periphery to core influences immune cell function, with glutamine competition between cancer cells and T cells inhibiting antitumor immune responses [274]. Mathematical modeling uncovered that cortisol-induced stress diminishes immune cell infiltration, with immune cell motility, infiltration capability, and growth rate being key determinants of infiltration patterns [276].

These spatial distribution patterns underscore critical barriers for immunotherapy efficacy in solid tumors. The limited penetration observed for T cells and NK cells directly affects adoptive cell therapies, as evidenced by chimeric antigen receptor T (CAR-T) cells showing restricted infiltration into the core of 3D breast cancer spheroids [277]. The immunosuppressive gradient established by M2-polarized macrophages in deeper spheroid regions may contribute to resistance mechanisms. Unlike small molecule drugs that rely primarily on passive diffusion through the ECM, immune cells require active migration and matrix remodeling capabilities, necessitating strategies to augment both physical penetration and functional persistence within the tumor microenvironment.

**Figure 3:**
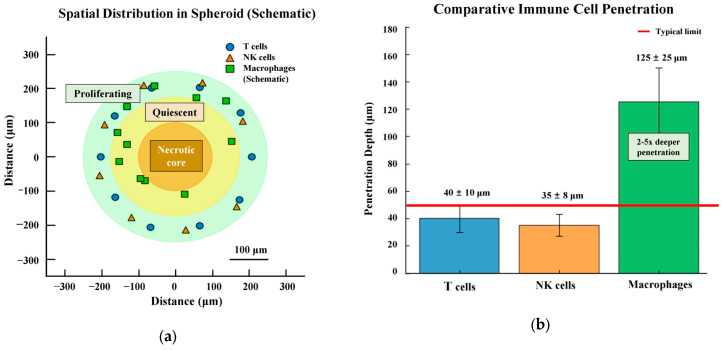
Immune cell distribution in 3D tumor spheroids. (**a**) Schematic representation of spatial distribution showing the characteristic peripheral localization of T cells and natural killer (NK) cells (30–50 μm penetration) with macrophages achieving deeper penetration (up to 125 μm) in a 500 μm diameter spheroid. Three distinct zones are depicted: proliferating rim (green outer circle), quiescent zone (yellow middle circle), and necrotic core (brown inner circle) (**b**) Comparative quantitative analysis of immune cell penetration depths. T cells and NK cells show limited penetration (40 ± 10 μm and 35 ± 8 μm, respectively), while macrophages demonstrate 2–5 × deeper penetration (125 ± 25 μm). The dashed line indicates the typical penetration limit (50 μm) for most immune cells. Data represent mean ± SD based on literature values from Section 4.2.1.

The spatial distribution of immune cells within tumor spheroids is illustrated in Figure 3. Panel a provides a schematic representation of the characteristic peripheral localization pattern, while Panel b quantitatively demonstrates the limited penetration depths observed across different immune cell types.

#### 4.2.2. Concentration Thresholds for Immunomodulatory Activity

Establishing minimum effective concentrations for immunomodulation in 3D systems uncovers substantial increases compared to 2D baselines. Curcumin requires higher concentrations for effective activity in spheroid cultures, with EC_50_ values increasing from 12.25 μM in 2D cultures to 30.76 μM in 3D spheroids, representing a 2.5-fold increase [60]. This observed change in drug response between 2D and 3D could be attributed to multiple factors including strong cell-to-cell interaction, compact cell packing, deposition of ECM between cells, or the existence of different cell layers within 3D tumor spheroid [278,279,280]. Similar patterns emerge across diverse compounds—estradiol-induced proliferation requires 15 nM in 2D systems but 50 nM in 3D spheroids to achieve comparable effects, demonstrating a 3.3-fold concentration increase [281]. Cyclopiazonic acid (CPA) shows IC_50_ values of 864 nM, 437 nM, and 392 nM in 2D cultures at 24, 48, and 72 h respectively, while corresponding 3D values are 1132 nM, 1069 nM, and 567 nM [282].

Dose–response curves indicate altered cooperativity in 3D systems, with Hill coefficients demonstrating reduced values compared to 2D cultures. In 3D spheroids, LNCaP cells possess a Hill coefficient of 1.252 while HepG2 cells present 2.807, indicating more gradual dose-dependent responses in LNCaP spheroids and implying different drug adsorption and delivery to single cells in tumor tissue [283]. The IC_50_ values are higher than 2D culture, suggesting that the structural hindrance of drug transport, delivery, and effect within 3D tumorous tissue models is primarily due to the ECM expression [279,284].

The area under the concentration-time curve (AUC_0→24_) requirements increase dramatically in 3D systems. For HeLa cells, AUC values must increase from 120 in monolayers to 480 in spheroids, while CAL-27 cells require an increase from 480 to 4800, representing 4-fold and 10-fold increases respectively [279]. Due to the introduction of the ECM, penetration of the drug is reduced [279,285].

Temporal dynamics add another layer of complexity, with immunomodulatory effects requiring sustained exposure exceeding those for direct cytotoxicity. Platycodin D downregulated PD-L1 expression on the surface of lung cancer cells with maximum effect at 6 h. After that, PD-L1 expression gradually recovered with further increases in treatment time, which may be due to cells maintaining cellular homeostasis through negative feedback mechanisms or adaptive responses [286]. Ki-67 expression, a proliferation marker, displays dose-dependent reduction after 72 h of continuous exposure, with all Pt compounds inhibiting the expression of the Ki-67 proliferation marker after 72 h in a dose-dependent manner [284,287].

The requirement for multiple treatment cycles becomes evident in 3D systems. Single-cycle treatments that effectively reduce viability in 2D cultures often fail to produce significant growth inhibition in 3D spheroids. Paclitaxel at 50 nM, which was well below the threshold of acute cytotoxicity on the basis of short-exposure experiments with microfluidic 2D cultures, yields no significant effect in 3D spheroids at the same timepoint [288]. Similarly, doxorubicin treatment attaining 66.98% cell death in 2D monolayers only produces 33.42% mortality in 3D spheroids after 48 h at identical concentrations [287]. Increased IC_50_ values in 3D cell culture system may be associated with multiple mechanisms, including a reduced penetration of chemotherapy agents because of the simulation of key factors of natural tumor microenvironment such as physiological gradients and presence of ECM, enhanced pro-survival signaling pathways [280,284,289].

The findings emphasize the importance of using 3D spheroids for drug screening due to the observed differences in cellular response between the 2D and 3D cell culture systems [278,281,290].

#### 4.2.3. Proposed Framework for Integrated Assessment

**Figure 4:**
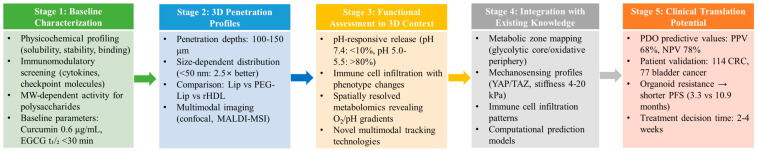
Five-stage evaluation framework for natural product-based immunomodulators in 3D spheroid models. Framework progresses from Stage 1 baseline characterization establishing drug properties, through Stage 2 3D penetration assessment, Stage 3 functional validation, Stage 4 integration with metabolic and immune patterns, to Stage 5 clinical translation using patient-derived organoids. Each stage provides critical data for optimizing the balance between penetration enhancement and activity preservation. The arrow (→) indicates a causal relationship where organoid resistance leads to shorter PFS. IL-6, interleukin-6; MALDI-MSI, matrix-assisted laser desorption/ionization mass spectrometry imaging; NF-κB, nuclear factor kappa B; PDO, patient-derived organoid; PD-L1, programmed death-ligand 1; PFS, progression-free survival; TNF-α, tumor necrosis factor-alpha.

The disconnect between penetration enhancement and immunomodulatory preservation necessitates new evaluation paradigms. We propose a comprehensive five-stage assessment framework that evaluates both spatial distribution and functional outcomes throughout the development pipeline (Figure 4).

Stage 1 establishes baseline immunomodulatory profiles in 2D systems, quantifying concentration-response relationships for key endpoints. Recent studies illustrate that natural products display diverse immunomodulatory mechanisms at varying concentrations [291,292,293]. For checkpoint molecules, synthetic PD-L1 inhibitors like BMS-202 show IC_50_ values of 18 nM, providing comparative benchmarks for natural product potency [294]. The programmed cell death protein 1 (PD-1)/PD-L1 axis represents a critical target, with natural products showing promise in modulating this pathway through both direct inhibition and expression regulation [294,295,296].

Cytokine production profiles reveal complex dose-dependent relationships. Tumor necrosis factor-alpha (TNF-α) stimulation induces IL-6 production in a dose-dependent manner (0–10 nanogram per milliliter (ng/mL)) [297], with synergistic effects observed when combined with metabolic stressors. Co-stimulation with TNF-α and stearate elevates IL-6 expression 81 ± 2.1-fold compared to 38 ± 0.5-fold with TNF-α alone, highlighting the importance of evaluating compounds under physiologically relevant inflammatory conditions [298]. These inflammatory cascades involve p38 MAPK signaling, with phosphorylation at threonine 180 and tyrosine 182 being critical for activity [297]. The relationship between TNF-α and IL-6 has been established in multiple contexts, including infectious diseases and inflammatory conditions [299,300].

Physicochemical characterization supplies essential baseline data for formulation development. Curcumin possesses poor aqueous solubility (0.6 μg/mL at pH 7.4) [301,302] and high albumin binding affinity (K = 1.04 − 2.0 × 10^5^ M^−1^), parameters that directly influence bioavailability and cellular uptake [303,304]. The binding occurs through hydrophobic interactions and follows 1:1 stoichiometry at the high-affinity site [303]. EGCG shows extreme instability in culture media with t_1_/_2_ < 30 min, generating H_2_O_2_ through auto-oxidation [305,306]. Temperature-dependent degradation follows first-order kinetics, with anthocyanins showing pH-dependent stability (t_1_/_2_ = 10.27 h at pH 3.0 vs. 4.66 h at pH 7.0 at 90 °C) [307,308]. These baseline characteristics guide subsequent formulation strategies and help predict behavior in 3D systems.

Natural polysaccharides exhibit molecular weight-dependent immunomodulatory profiles. Polysaccharides with molecular weights exceeding 1000 dalton (Da) have been reported to elicit immunostimulatory effects in macrophages [309]. High molecular weight polysaccharides (>100 kDa) from various sources including *Antrodia cinnamomea* have shown potent activation of dendritic cells and macrophages through TLR4-dependent pathways [310,311]. However, the relationship between molecular weight and biological activity varies with the source and target activity, as evidenced by β-glucans from Qingke where lower molecular weights presented enhanced anti-cancer effects while higher weights favored anti-inflammatory activity [312]. These findings underscore the complexity of polysaccharide structure-activity relationships and the importance of preserving structural integrity during formulation. Together, these baseline characterizations—from small molecule physicochemical properties to macromolecular structure-function relationships—established essential parameters for evaluating drug behavior in 3D systems, leading to Stage 2 penetration studies.

Stage 2 focuses on quantifying penetration depths (typically 100–150 μm) and elucidating formulation-dependent distribution mechanisms through three complementary approaches.

Optical imaging techniques supply real-time penetration mapping. Confocal microscopy tracks fluorescent drug distribution throughout spheroids [313], while light-sheet microscopy uncovers size-dependent penetration mechanisms: particles < 50 nm attain 2.5-fold better penetration than larger particles due to reduced steric hindrance in the extracellular matrix. Specifically, 20–50 nm particles diffuse through interstitial spaces to reach cores, whereas 100 nm particles become trapped in peripheral cell layers [6,94]. Quantitative analysis of these imaging data can be performed using automated tools like INSIDIA macro, facilitating high-throughput characterization of spheroid invasion parameters [314].

Chemical detection methods quantify drug concentrations spatially. Matrix-assisted laser desorption/ionization mass spectrometry imaging (MALDI-MSI) achieves 5–10 μm resolution for label-free drug mapping, revealing that irinotecan (20.6 μM, 48h) reaches 16.90 μM in cores—evidence of efficient penetration despite concentration gradients [315,316,317]. These measurements occur within spheroids’ characteristic microenvironment: three distinct zones (proliferative periphery, quiescent middle, necrotic core) with hypoxia beyond 200 μm and pH gradients from 7.4 → 6.7–6.9 that affect drug stability and cellular uptake [6,318,319,320,321].

Penetration mechanisms vary dramatically with formulation. Lipid-based nanoparticles show distinct patterns: conventional liposomes bind extensively to peripheral cells, PEGylated liposomes reduce this binding through steric shielding (78% remaining interstitial), while reconstituted high-density lipoprotein (rHDL) exploits endogenous transport pathways for exceptional core accumulation [6,48,262,322]. Positive charges trap particles peripherally via electrostatic adhesion, while neutral/negative surfaces permit deeper diffusion [6,196]. Flow dynamics also matter—higher velocities (96 vs. 19 micrometer per second (μm/s)) augment penetration through convective transport [323].

Emerging approaches address current limitations. Tissue-clearing (ClearT/ClearT2) enhances imaging depth without sectioning [324], while protein-disrupting drugs allow real-time tracking without labels [325]. These innovations complement established techniques, providing unprecedented insights into penetration dynamics.

This multifaceted characterization indicates that effective penetration requires optimizing particle size (<50 nm), surface chemistry (neutral/slightly negative), and delivery conditions (flow-enhanced transport). However, single-technique analyses offer limited perspectives—comprehensive understanding demands integrated multimodal approaches, as explored in Stage 3.

Stage 3 evaluates whether enhanced penetration translates to preserved biological activity within the 3D microenvironment. pH-responsive release mechanisms demonstrate controlled drug liberation in acidic tumor compartments, with polymer-drug conjugates showing minimal release (<10%) at physiological pH 7.4 but rapid release (> 80%) at endosomal pH 5.0–5.5 [48,326]. This pH-triggered release occurs through hydrazone bond cleavage, ensuring drug activation specifically within target cells rather than during transit [326]. The spatial distribution of drug metabolites, visualized through MALDI-MSI, confirms that active compounds reach deeper spheroid regions while maintaining chemical integrity [317].

Immunomodulatory activity assessment exposes complex spatial patterns within spheroids. Natural killer (NK) cells show active infiltration through 3D matrices, with maintained cytotoxic function against embedded tumor cells [327]. Real-time imaging captures NK cell extravasation and migration through collagen barriers, with decreased CD16-positive NK cells within the infiltrated population [327]. CAR-T cells similarly penetrate spheroid structures and excert cytotoxic effects [274,277], though penetration depth correlates inversely with target antigen density on peripheral cells [277]. This “antigen sink” effect emphasizes the importance of spatial considerations in immunotherapy design [277].

Metabolic reprogramming accompanies immune cell infiltration, as uncovered by spatially resolved metabolomics [274]. Beyond glutamine alterations, spatial heterogeneity in both glycolytic and oxidative phosphorylation pathways correlates with local oxygen concentrations [328]. Live imaging through phosphorescence lifetime imaging microscopy discloses O_2_ microgradients that drive metabolic zone formation [329], while lactate accumulation reaching up to 40 mM in spheroid cores generates metabolic stress influencing immune cell function [330]. The combination of berberine treatment with immune cell co-culture illustrates how small molecules can modulate both tumor metabolism and immune function simultaneously [274].

The tumor microenvironment presents additional barriers through extracellular matrix components. Tissue inhibitor of metalloproteinases-1 (TIMP-1) expression by cancer-associated fibroblasts forms dense matrix barriers that limit both drug and immune cell penetration [17]. However, modifications to delivery systems can improve penetration through these barriers, as shown by size-dependent polyplex penetration in spheroids [196]. These findings emphasize the dynamic nature of the 3D microenvironment and the need for formulations that can adapt to or modify these barriers.

Novel tracking methodologies enable unprecedented insights into spatiotemporal drug behavior. Beyond fluorescent protein disruption [325], emerging techniques include gadolinium-based magnetic resonance imaging (MRI) for real-time spatial imaging [331], fluorescence lifetime imaging microscopy for metabolic state assessment [329], and microfluidic platforms permitting continuous real-time observation of drug-spheroid interactions [332]. These multimodal approaches reveal distinct penetration profiles for different formulations with unprecedented resolution, supplying valuable predictive tools for formulation optimization.

Stage 4 synthesizes experimental findings with computational modeling to predict optimal formulation parameters. The 3D spheroid metabolic profile differs significantly from 2D cultures, with enhanced glycolytic activity and altered oxidative phosphorylation patterns [333]. Adenosine triphosphate (ATP)-linked respiration increases in spheroids under standard nutrient conditions, while glycolytic capacity rises under glucose-depleted conditions, revealing metabolic flexibility [333,334]. This metabolic reprogramming directly affects drug response, as spheroids with higher glycolytic activity display increased resistance to certain therapeutics [333].

Spatial metabolomics reveals heterogeneous metabolic zones within spheroids. Nicotinamide adenine dinucleotide phosphate (NADP(H)) autofluorescence imaging shows formation of glycolytic cores surrounded by oxidative phosphorylation-active peripheries in some spheroid types, producing “inverted” oxygen gradients contrary to traditional assumptions [329]. These metabolic gradients influence both drug penetration and immunomodulatory activity distribution. Size-dependent metabolic shifts occur at approximately 300–400 μm diameter, where hypoxic cores develop and metabolic enzyme expression patterns change [280,329].

Mechanical properties of the 3D environment profoundly influence cellular responses and drug efficacy. Spheroids cultured in matrices with varying stiffness (4–20 kPa) exhibit differential drug penetration and cellular mechanosensation [335,336]. Yes-associated protein (YAP)/transcriptional coactivator with PDZ-binding motif (TAZ) mechanotransduction pathways respond differently in 3D versus 2D, with nuclear localization suppressed in spheroids despite increased cell spreading [337]. This altered mechanosensing influences proliferation rates and drug sensitivity, requiring formulation strategies that account for matrix mechanical properties.

Integration of immune components discloses complex spatial relationships. Tumor-associated macrophages (TAMs) infiltrate spheroids with patterns dependent on both spheroid composition and inflammatory cytokine profiles [271,338]. Co-culture spheroids containing cancer cells and fibroblasts secrete distinct cytokine profiles, with IL-6 and TNF-α levels varying by cell line and modulating monocyte recruitment and polarization [271]. Natural killer cell infiltration follows similar spatial patterns, with CD16 expression changing during migration through 3D matrices [327].

Advanced computational approaches facilitate prediction of optimal delivery strategies. Machine learning algorithms trained on spheroid penetration data can predict formulation performance based on physicochemical properties including size, charge, and hydrophobicity, as shown in recent computational studies. Integration of spatial transcriptomics with drug distribution mapping reveals gene expression changes correlating with local drug concentrations [339]. These multi-scale models incorporate diffusion-reaction equations with cellular uptake kinetics to predict spatiotemporal drug distribution patterns. These integrated findings from model systems required validation in clinically relevant contexts, leading to Stage 5 patient-derived studies.

Stage 5 evaluates the translational relevance of 3D spheroid findings for clinical applications. Patient-derived organoids (PDOs) demonstrate remarkable correlation with clinical treatment outcomes, achieving 68% positive predictive value and 78% negative predictive value for treatment response prediction [340]. This outperforms empirically guided treatment selection, highlighting the clinical utility of 3D models for personalized medicine approaches [340]. The correlation between organoid and patient responses strengthens when culture conditions closely mimic physiological environments, particularly using human plasma-like medium (HPLM) versus standard culture media [341,342].

Drug resistance mechanisms observed in spheroids accurately reflect clinical resistance patterns. ATP-binding cassette subfamily B member 1 (ABCB1) upregulation (92–118 fold) in dabrafenib-resistant colorectal cancer spheroids and ATP-binding cassette subfamily G member 2 (ABCG2) elevation in irinotecan-resistant models mirror clinical chemoresistance phenotypes [343]. These resistance mechanisms develop through both pre-existing and de novo pathways, as disclosed by cellular barcoding technology tracking clonal evolution [343]. The polyclonal nature of resistance observed in spheroids recapitulates the heterogeneous resistance patterns seen in patient tumors.

Clinical biomarker validation shows strong concordance between spheroid models and patient outcomes. Excision repair cross-complementation group 2 (ERCC2) mutations predict cisplatin sensitivity in both bladder cancer organoids and patients (*p* = 0.002), while Kirsten rat sarcoma viral oncogene homolog (KRAS) mutations correlate with mitogen-activated protein kinase (MEK) inhibitor resistance [344,345]. Network-based machine learning approaches using organoid pharmacogenomic data attain high accuracy in predicting patient responses to 5-fluorouracil (114 colorectal cancer patients) and cisplatin (77 bladder cancer patients) [344]. These predictions outperform traditional cell line-based models in identifying clinically relevant biomarkers.

Practical implementation considerations expose both opportunities and challenges. Culture success rates vary significantly between tumor types, with optimized protocols reaching 55% success for high-grade serous ovarian cancer versus 23–38% with standard methods [342]. Cryopreserved tissue permits organoid derivation, expanding biobanking possibilities [342]. However, approximately 30% of patients experience clinical deterioration during standard care before organoid-guided treatment can be implemented [346].

Recent advances illustrate successful prediction of responses to targeted therapies. PDOs accurately predicted clinical responses to osimertinib in epidermal growth factor receptor (EGFR)-mutant lung cancer and identified effective combination therapies for resistance mutations (EGFR/B-Raf proto-oncogene (BRAF) co-mutations responding to dabrafenib/trametinib) [347]. The ability to screen multiple drugs simultaneously on limited patient material yields actionable information within clinically relevant timeframes (typically 2–4 weeks) [346,347].

### 4.3. Critical Challenges and Future Directions

Despite the comprehensive framework presented above, several critical challenges impede clinical translation. First, the fundamental penetration–activity trade-off remains unresolved, with no current formulation strategy successfully maintaining full biological activity while achieving deep tissue penetration. Second, the lack of standardized protocols prevents meaningful comparison across laboratories, as evidenced by the recent need for initiatives like the Organoid Standards Initiative [348]. Third, the cost and complexity of patient-derived organoid models, with establishment success rates varying from 20–85% [349,350], limit widespread adoption. Future perspectives should focus on paradigm shifts rather than incremental improvements. AI-driven optimization, which has achieved 93.44% accuracy in drug concentration prediction [351], could identify novel formulation strategies beyond current capabilities. Development of hybrid 3D models incorporating perfusion and immune components [33,327] will provide more predictive platforms. For the pharmaceutical industry, this framework offers transformative potential in natural product development. The systematic optimization of the penetration–activity balance enabled by 3D models addresses a critical bottleneck where promising immunomodulatory natural products fail during formulation development. By providing predictive platforms for evaluating formulation strategies before costly clinical trials, pharmaceutical companies can rescue previously abandoned natural product candidates and significantly reduce development timelines. The standardized protocols and regulatory guidance proposed here directly support industry needs for reproducible, FDA-acceptable screening methods. We recommend immediate establishment of consortium-based validation studies, development of open-access databases, and regulatory guidance building on FDA Modernization Act 2.0 [352]. These coordinated efforts are essential for translating natural product-based immunotherapies from bench to bedside.

## 5. Model System Variations and Their Impact

### 5.1. Analysis of 3D Culture Platforms

#### 5.1.1. Spheroid Models: Advantages and Limitations

Spheroid models represent the most widely adopted 3D platform for natural product penetration studies, offering reproducibility and scalability advantages. Spheroid size-related IC_50_ values can vary up to 160% in anticancer drug treatments [353], and larger spheroids with greater complexity exhibit enhanced drug resistance due to ECM barriers and efflux pump upregulation [354]. This presents a paradox where more physiologically relevant models may underestimate therapeutic potential, as drug resistance increases with spheroid complexity and ECM deposition. The choice of spheroid production method significantly influences drug resistance properties [354], necessitating standardized approaches for reproducible results. Furthermore, patient-derived organoids have emerged as valuable preclinical models, with studies confirming that organoids can accurately predict individual drug responses for personalized medicine [343,355,356]. This evolution from standardized spheroid models to patient-specific organoid systems represents a critical advancement in bridging the gap between in vitro testing and clinical outcomes.

#### 5.1.2. Organoid Systems: Patient-Specific Considerations

While spheroid models provide valuable insights into basic 3D drug penetration mechanisms, patient-derived organoids represent a more physiologically relevant yet complex system for evaluating natural product delivery. Unlike standardized cell lines used in spheroids, organoid establishment itself exhibits remarkable heterogeneity, with success rates varying from 20% in gastric cancer to 85.7% in drug-untreated breast cancer patients [349,350]. This variability extends beyond establishment to fundamental drug response patterns, where individual patient-derived organoids display differential sensitivities even to identical compounds [357].

While spheroid models provide valuable insights into basic 3D drug penetration mechanisms, patient-derived organoids represent a more physiologically relevant yet complex system for evaluating natural product delivery. Patient heterogeneity manifests at multiple levels that directly impact natural product efficacy. Organoid drug responses show moderate but variable correlation with clinical outcomes: 0.58 for 5-fluorouracil (5-FU), 0.61 for irinotecan, and 0.60 for oxaliplatin-based therapies [341]. This variability is compounded by ECM composition differences between patients, where TIMP-1 expression increases ECM deposition and reduces drug penetration in breast cancer spheroids [17]. For natural products, this heterogeneity is particularly challenging as compounds like flavonoids must maintain their structural integrity while navigating patient-specific barriers. Polyphenol natural products illustrate this complexity, requiring innovative delivery strategies to preserve both penetration capability and bioactivity [358].

The scaffold-dependent nature of organoid culture further complicates penetration assessment. Matrigel, containing approximately 60% laminin, creates an artificial barrier that may not accurately represent patient tumors [359]. Different basement membrane extract (BME) sources (Matrigel, Cultrex, UltiMatrix) influence organoid proliferation but not drug response curves [360], suggesting that penetration barriers are more dependent on organoid-intrinsic factors than scaffold composition. This finding has important implications for natural product screening, as demonstrated by flavonoids’ successful activity against severe acute respiratory syndrome coronavirus 2 (SARS-CoV-2) in lung organoids despite the Matrigel barrier [361], indicating that bioactivity can be maintained even with compromised penetration.

Emerging technologies offer solutions to address patient-specific variability in natural product testing. Automated microfluidic platforms achieve inter-organoid homogeneity while maintaining inter-patient heterogeneity, with 97% retention of parental tumor mutations [362]. Alternative matrices like decellularized intestine ECM provide more physiologically relevant microenvironments [363], potentially offering better predictive value for natural product penetration. Additionally, suspension culture techniques with reduced ECM concentration (5% vs. 80%) maintain organoid characteristics while improving screening efficiency, enabling more accurate assessment of penetration-limited compounds [364].

The implications for natural product development are significant. Matrix stiffness modulates drug response, with stiffer matrices conferring increased resistance [365], while organoid size, shape, and growth patterns create additional heterogeneity [366]. Single-cell analysis uncovers drug-resistant subpopulations within individual organoids [367], suggesting that natural products must overcome not only physical barriers but also cellular heterogeneity. These findings underscore the need for patient-specific formulation strategies that account for both penetration barriers and biological variability when developing natural product-based therapeutics, pointing toward the necessity of advanced scaffold designs and microfluidic technologies that can better recapitulate and control these complex microenvironments, as discussed in the following section. Although this review focuses on tumor organoids for evaluating natural product penetration in cancer, organoid technology extends beyond oncology to model various diseases including inflammatory bowel disease, cystic fibrosis, infectious diseases, and neurological disorders [368,369,370,371,372]. However, the unique penetration challenges in tumor microenvironments—particularly the dense ECM and hypoxic gradients—make tumor organoids especially relevant for studying the penetration–activity trade-off of natural products.

#### 5.1.3. Scaffold-Based and Microfluidic Innovations

While spheroids and organoids provide valuable insights into natural product penetration, scaffold-based and microfluidic models offer unique advantages for understanding the complex physicochemical interactions between natural products and tissue architecture. These advanced platforms are particularly crucial for natural products, which often exhibit distinct penetration patterns due to their diverse chemical structures, including glycosidic moieties that increase solubility and change bioavailability of their aglycones [373].

Scaffold-based models introduce architectural features that specifically challenge natural product delivery. Collagen scaffolds with defined pore sizes (68–121 μm) reveal how natural products exhibit bimodal distribution patterns—a phenomenon particularly relevant for multi-component botanical extracts [374,375]. This bimodal pattern arises from the heterogeneous nature of natural products: hydrophilic glycosidic components rapidly diffuse through scaffold pores, while hydrophobic aglycones show restricted penetration into scaffold struts where cells preferentially attach [373,376]. For natural products rich in hydrophobic compounds like curcumin and quercetin, silk fibroin scaffolds present particular challenges. The hydrophobic amino acids in silk fibroin structure significantly impair water-soluble formulation penetration, with swelling capacity reduced compared to hydrophilic materials, directly impacting the delivery of water-soluble natural product preparations [377,378]. This effect was quantified in gelatin-based scaffolds where higher swelling degrees increased gel layer thickness and reduced natural product release rates by 41–42% in the first few hours—a critical consideration for time-sensitive bioactive compounds [379].

Microfluidic platforms revolutionize natural product penetration assessment by recapitulating the dynamic physiological processes that significantly impact botanical therapeutics. For natural products, these systems provide unprecedented insights into how traditional preparations are metabolized. Tumor-on-chip devices illustrate that natural product nanoformulations achieve 3–20 fold enhanced penetration under flow conditions compared to static cultures, with optimal shear stress of 2.5 dyne per square centimeter (dyne/cm^2^) maintaining endothelial barrier function while enabling controlled extravasation [380,381]. Most critically for natural products, multi-organ microfluidic systems reveal the dramatic impact of first-pass metabolism—a phenomenon that has historically limited oral bioavailability of many botanical therapeutics [379]. Liver-on-chip systems revealed that natural products undergo extensive hepatic metabolism, with compounds like midazolam (often used as a probe for herbal-drug interactions) showing complete metabolism within minutes (t½ = 2.7 min (min)), while intestinal metabolism proceeded more slowly (t½ = 157–198 min) [382,383]. In gut-liver axis chips, up to 70% of orally administered natural products accumulated in the intestinal compartment, explaining the traditionally poor systemic bioavailability of many botanical medicines, which typically show only about 6% oral bioavailability [379,384,385].

These scaffold and microfluidic models thus provide essential tools for optimizing natural product formulations, with organ-on-chip technology now gaining regulatory acceptance and pharmaceutical industry adoption for drug screening and development [386,387], offering mechanistic insights that guide the development of delivery strategies to overcome the unique penetration barriers faced by botanical therapeutics. The integration of these platforms enables researchers to move beyond empirical observations toward rational design of natural product delivery systems. Building on these mechanistic insights, the following section explores specific formulation approaches that have been developed to enhance natural product penetration through these identified barriers.

**Figure 5:**
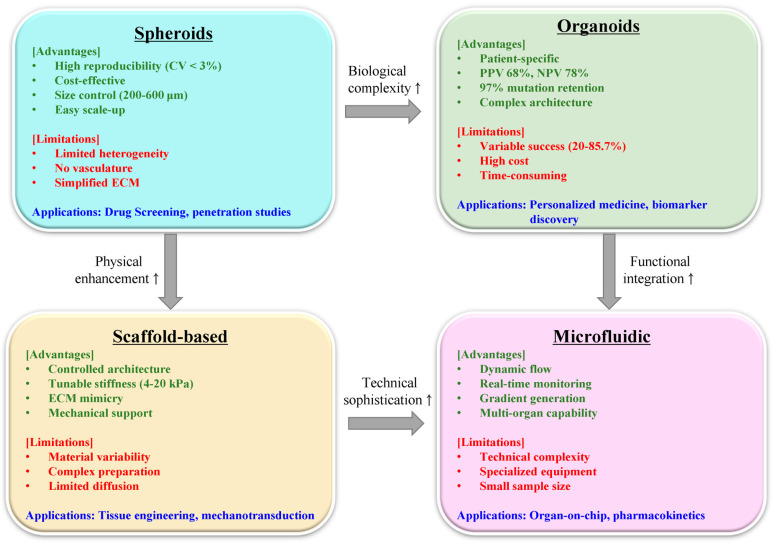
Comparison of 3D model platforms for natural product testing. Overview of advantages (green), limitations (red), and applications (blue) for spheroids, organoids, scaffold-based, and microfluidic systems. Arrows (↑) indicate increase or enhancement relative to 2D culture systems. Success rates and predictive values are indicated at the bottom. CV, coefficient of variation; PPV, positive predictive value; NPV, negative predictive value; ECM, extracellular matrix.

These diverse 3D culture platforms offer complementary advantages for natural product testing, as summarized in Figure 5. Selection of the appropriate platform depends on research objectives, with spheroids providing cost-effective initial screening, organoids enabling personalized medicine approaches despite variable establishment success (20–85.7%), scaffold-based systems facilitating mechanotransduction studies, and microfluidic platforms allowing dynamic drug testing with real-time monitoring capabilities.

Building on these microfluidic advances, organ-on-chip technology represents the next evolutionary step, integrating multiple tissue types with perfusion systems to create more physiologically relevant models. The manufacturing of these microfluidic devices typically employs soft lithography techniques using PDMS, creating precise channel geometries (100–500 μm) that enable controlled flow rates and gradient generation, as detailed in recent reviews on 3D in vitro models [388]. Recent advances in 3D printing techniques, particularly stereolithography and bioprinting, provide alternative fabrication methods for these complex systems [389]. These platforms offer superior control over fluid dynamics and cellular microenvironments compared to static 3D models, though their complexity and cost currently limit widespread adoption for natural product screening. Future integration of organ-on-chip technology with the formulation strategies discussed herein could bridge the gap between enhanced penetration and preserved bioactivity through real-time monitoring of drug-tissue interactions.

### 5.2. Recent Technological Advances

#### 5.2.1. Vascularized Models for Evaluating Penetration–Activity Balance

While previous sections established the penetration–activity trade-off in static 3D models, vascularized tumor models add another layer of complexity that particularly affects natural product therapeutics. Unlike static spheroids where diffusion is the primary transport mechanism, vascularized models introduce convective flow, creating dynamic microenvironments that can either enhance or compromise the delicate balance between natural product penetration and bioactivity [6,390].

The presence of functional vasculature fundamentally alters the penetration–activity equation for natural products. Natural products face unique challenges in vascularized systems due to their metabolic susceptibility. Endothelial cells lining the vasculature express metabolizing enzymes including sulfotransferases that can modify polyphenols during transcapillary transport [391,392]. This metabolism during transit represents an additional mechanism of activity loss not present in static models. Furthermore, the oxygen gradients established by vascular perfusion—ranging from normoxic at vessel walls to hypoxic in avascular regions—create zones where natural products undergo differential degradation, as illustrated for anthocyanins and EGCG which show pH- and oxygen-dependent stability [393,394].

The vascular normalization potential of natural products presents both opportunities and challenges for the penetration–activity balance. Compounds like ginsenoside Rg3 and resveratrol can normalize tumor vasculature, potentially improving their own delivery in subsequent doses [395,396]. Low-dose anti-angiogenic treatments have been shown to induce vascular normalization windows lasting 3–6 days, during which improved drug delivery can be achieved [397,398]. However, this normalization process requires natural products to maintain bioactivity during initial penetration through abnormal vessels [399].

The complex interplay between formulation design and vascular dynamics requires careful consideration in perfused systems. Nanoparticle systems that excel in static spheroids may fail in perfused systems due to rapid clearance or vascular margination. Studies using continuously perfused microbubble arrays indicates that flow characteristics and shear stress profiles significantly affect drug distribution, with different flow velocities creating distinct drug accumulation patterns [400]. The binding-site barrier effect is amplified in vascularized models, where high-affinity targeting can trap natural products in perivascular regions, preventing deeper penetration while potentially losing activity through prolonged vascular residence [401].

Oxygen dynamics in vascularized models create spatial heterogeneity affecting natural product stability and activity. The lateral oxygen gradients observed in vascularized tumor models, where oxygen consumption creates centripetal gradients from vessel walls, establish zones of differential natural product behavior [394]. Mathematical modeling of angiogenic factor distribution shows that interstitial fluid pressure significantly affects the balance of pro- and anti-angiogenic factors, creating regions where natural products may be more or less stable [402]. These spatial variations in stability create a complex landscape where penetration depth does not directly correlate with therapeutic efficacy.

Current research reveals a critical gap in understanding how vascularization affects the penetration–activity balance for natural products. While studies confirm enhanced delivery through vascular networks [393,403], parallel assessment of activity preservation remains absent. Multi-organ-on-chip systems, such as the hollow fiber membrane-based liver organoid model revealing 70% accumulation of natural products in intestinal compartments with only 6% systemic bioavailability, highlight how vascular compartmentalization can sequester compounds away from target sites [383]. This spatial segregation, combined with metabolic losses during vascular transit, suggests that the penetration improvements in vascularized models may not translate to enhanced therapeutic efficacy.

Thus, vascularized models reveal that the penetration–activity balance for natural products is further complicated by dynamic blood flow, oxygen gradients, and metabolic factors unique to perfused systems. Future research must move beyond simple penetration measurements to assess spatiotemporal activity preservation, developing integrated metrics that capture both enhanced delivery and metabolic losses in vascularized tumor microenvironments. Only through such comprehensive evaluation can formulation strategies be optimized to exploit the benefits of vascular delivery while preserving the therapeutic activity of natural products.

#### 5.2.2. Real-Time Monitoring Technologies

Advanced microscopy techniques enable real-time visualization of drug penetration dynamics in 3D tumor models. Lattice light-sheet microscopy achieves spatial resolution of 235 nm × 235 nm × 340 nm, allowing visualization of drug distribution at sub-second intervals [404]. Two-photon light-sheet nanoscopy provides complementary approaches with enhanced spatiotemporal resolution through fluorescence fluctuation analysis [405]. This high spatiotemporal resolution is crucial for capturing rapid transport processes that occur during initial drug exposure.

The temporal resolution of modern tracking systems has reached unprecedented levels. Three-dimensional orbital tracking microscopy achieves nanometer precision with millisecond temporal resolution, enabling continuous monitoring of drug carriers over distances exceeding 100 μm [406]. Similar millisecond-scale tracking has been accomplished for mitochondrial dynamics in four-dimensional (4D) imaging systems [407]. These capabilities allow researchers to distinguish between different transport mechanisms in real-time, including passive diffusion and active cellular processes.

Active transport mechanisms significantly enhance drug penetration compared to passive diffusion alone. Tumor-penetrating peptides utilizing transcytosis pathways achieve up to 300% enhancement in penetration depth [408]. The iRGD peptide system produces 250% increase in doxorubicin penetration through C-end rule (CendR)-mediated transport [409]. Additionally, reduction of cancer-associated fibroblast activity decreases extracellular matrix density, further facilitating nanoparticle penetration into deeper tumor regions [149].

Metabolic processes and drug distribution can be monitored using coherent anti-Stokes Raman scattering (CARS) microscopy. This label-free technique enables quantitative measurement of glucose concentrations at the single-cell level [410] and detection of C-deuterated drugs with millimolar sensitivity [411]. CARS microscopy has been successfully applied to visualize paclitaxel distribution in polymer films with 0.3 μm lateral resolution [412], providing insights into drug release kinetics without fluorescent labeling.

Imaging depth remains a critical parameter for comprehensive tumor analysis. Two-photon microscopy typically achieves penetration depths of 100–300 μm depending on tissue properties and excitation wavelength [413,414]. Third-harmonic generation microscopy extends this capability to 420 μm, representing a three-fold improvement over conventional two-photon imaging [415]. Adaptive optics correction further enhances both imaging depth and resolution by compensating for tissue-induced aberrations [416,417]. These enhanced penetration depths enable visualization of drug distribution throughout entire tumor spheroids, including hypoxic core regions where drug resistance often develops [418].

#### 5.2.3. Multi-Scale Integration for Comprehensive Immune Monitoring

The integration of spatial and temporal heterogeneity with single-cell functional analysis presents new opportunities for understanding immune responses in complex tissue environments. Recent advances in analytical technologies enable unprecedented multi-scale characterization of immune cell behavior and function.

Advanced microfluidic platforms have achieved remarkable sensitivity for immune monitoring. Microfluidic enzyme-linked immunosorbent assay (ELISA) systems exhibit detection limits ranging from 0.021 to 20 picogram per milliliter (pg/mL) for various cytokines, with IL-6 detection as low as 0.021 pg/mL, prostate-specific antigen (PSA) at 0.047 pg/mL, and carcinoembryonic antigen (CEA) at 0.29 pg/mL [419]. This represents over 200-fold improvement compared to commercial ELISA methods, enabling detection of low-abundance immune mediators critical for understanding cellular communication networks [420].

Spatial resolution has dramatically improved through microfabricated sampling technologies. Push-pull sampling probes achieve spatial resolution of 20 × 60 μm, approximately 1000-fold better than conventional microdialysis probes with 2 mm long × 220 μm diameter membranes [421]. Alternative approaches using graphene microtransistors provide 50 × 50 μm spatial resolution, enabling nearly cellular-scale monitoring of neurochemicals and potentially immune mediators [422]. These advances allow precise mapping of local immune microenvironments and cytokine gradients.

The importance of spatial heterogeneity is evident in tumor microenvironments, where distinct gradients exist between core and peripheral regions. Studies reveal that tumor cores exhibit pronounced hypoxic conditions compared to the periphery, with hypoxia-inducible factor (HIF) activity showing radial increases from periphery to center [423]. This spatial organization correlates with differential cytokine production patterns, as hypoxic conditions are known to modulate immune cell function and cytokine secretion profiles [424,425,426].

Mass cytometry (CyTOF) has emerged as a powerful tool for high-dimensional immune profiling. Using panels of 30–34 markers, CyTOF can identify at least 8 major immune cell populations in complex tissues [427]. Moreover, detailed analysis uncovers functional heterogeneity within traditionally defined populations, such as the identification of 4 distinct subpopulations within phenotypically basophilic granulocytes [428]. This level of resolution is crucial for understanding how spatial location influences immune cell phenotype and function.

Integration of these multi-scale approaches exposes complex relationships between spatial location, cellular phenotype, and functional capacity. For instance, the combination of high-resolution spatial sampling with multiplexed cytokine analysis enables correlation of local microenvironmental conditions with immune cell activation states [429]. This comprehensive approach is essential for developing effective immunotherapies that account for both spatial and functional heterogeneity in complex tissue environments.

## 6. Emerging Technologies and Future Directions

### 6.1. Clinical Translation Pathways

#### 6.1.1. Regulatory Considerations and Standards

The path from promising 3D model results to clinical application requires navigation of evolving regulatory frameworks. The FDA Modernization Act 2.0, enacted in December 2022, has created unprecedented opportunities for organoid-based drug development by eliminating mandatory animal testing requirements and promoting the adoption of New Approach Methodologies (NAMs), including organoids and organ-on-chip technologies [352,430]. This legislative change reflects growing confidence in 3D models as acceptable alternatives for preclinical drug testing, with the FDA specifically mentioning these models as being acceptable in drug filing for market approval [431]. For natural product formulations, this regulatory shift is particularly significant given the complex nature of botanical therapeutics that interact with biological systems through multiple pathways [432].

Recognizing the need for standardization, various initiatives have emerged globally. In Europe, drug testing using organoids must comply with the European Medicines Agency’s generic “Guideline on the principles of regulatory acceptance of 3Rs (replacement, reduction, refinement) testing approaches” [433]. Korea established the Organoid Standards Initiative in September 2023, bringing together industry, academia, regulatory agencies, and standard development experts through public–private partnerships [348]. This initiative developed general guidelines for organoid manufacturing and quality evaluation (v1.0), providing guidance on materials, procedures, and essential quality assessment methods applicable at current technology levels [348]. However, regulatory harmonization remains a significant challenge, as evidenced by divergences in biowaiver criteria, dissolution profile requirements, and coefficient of variation standards across different countries [434].

Quality metrics for natural product formulations in 3D systems require adaptation from traditional pharmaceutical standards. Recent advances in automated 3D bioprinting have yielded remarkable reproducibility, with coefficient of variation (CV%) of embedded spheroid diameter between 4.2% and 8.7%, comparable to values obtained from ultra-low attachment 96-well plates [435]. Standardized mean difference (SSMD) analysis confirms that 95% of printed embedded spheroids meet “good or greater quality” criteria [435]. Automated platforms can now produce over 50 uniform spheroids with consistent size and circularity in 30 min with over 97% sorting accuracy [436], illustrating the feasibility of high-throughput standardized production for natural product screening. Despite these advances, challenges persist including high inter-lab variability and the need for standardization in spheroid generation methods [436].

Content uniformity poses particular challenges when drug distribution is inherently heterogeneous in 3D systems. Complex in vitro models positioned for pharmaceutical testing require assessment approaches that consider the unique characteristics of 3D environments and establish performance criteria for specific test assays [437]. Zone-specific evaluation can be performed at defined depths of 25, 50, 75, and 100 μm to assess penetration patterns [324]. For immunomodulatory natural products, functional assessment becomes crucial, as these compounds display antioxidant, antimicrobial, and immunomodulatory activities that must be preserved during formulation [438]. Automated high-throughput screening platforms now enable detailed analysis within 3D structures, allowing correlation of drug distribution with biological activity [436]. This integrated approach is essential for natural products where the preservation of biological activities is paramount.

The regulatory landscape continues to evolve through adaptation of existing frameworks. The FDA’s product-specific guidance (PSG) program provides recommendations for bioequivalence studies that could serve as a template for developing 3D model-specific standards [439]. The f2 similarity factor (≥ 50) used in dissolution profile comparisons represents one area where international harmonization is needed [434]. For natural product formulations optimized in 3D tumor models, establishing standardized protocols that balance scientific rigor with practical feasibility while accounting for the complex pharmacological profiles of botanical therapeutics will be essential for successful clinical translation. While specific regulatory guidelines for natural product formulations in 3D models are still evolving, the current framework provides a foundation for future development.

#### 6.1.2. Integrating Formulation Strategies with 3D Model Validation

The inherent challenges of natural product delivery—particularly the trade-off between penetration depth and bioactivity preservation—have prompted the development of advanced formulation strategies validated through 3D tumor models. Recent studies establish that appropriately designed nanoformulations can overcome these limitations while maintaining therapeutic efficacy.

Penetration enhancement through formulation optimization has shown remarkable progress. iRGD-modified nano-delivery systems based on marine sulfated polysaccharides exhibited significantly improved tumor targeting and cellular internalization, with the iRGD peptide facilitating deeper tissue penetration through neuropilin-1 (NRP-1) receptor-mediated transcytosis [133]. Similarly, nanoemulsions with particle sizes below 100 nm accomplished 24-h cumulative percutaneous permeation in 3D skin models, with the smaller particle size proving crucial for stratum corneum penetration [440]. These formulations maintained their bioactivity, as evidenced by paeonol/madecassoside co-delivery nanoemulsions (PM-NEs) that significantly promoted the secretion of filamentous protein, aquaporin 3 (AQP3), and Claudin-1 in 3D epidermal models while inhibiting inflammatory mediators including IL-1α, IL-6, and TNF-α [440].

The integration of 3D models for formulation validation has proven essential for predicting clinical outcomes. Patient-derived organoids achieved positive predictive values of 68% and negative predictive values of 78% for standard chemotherapy responses [340], while machine learning approaches applied to these 3D models reached 68.75% accuracy with random forest classifiers in predicting drug responses [441]. Notably, 3D spheroid models uncovered penetration depths ranging from 40–150 μm correlating with clinical efficacy, with doxorubicin showing a characteristic penetration depth of 40–50 μm associated with therapeutic response [442].

Advanced formulation strategies have successfully addressed the penetration-bioactivity trade-off. Deep eutectic solvent self-assembled reverse nanomicelles (DES-RM) demonstrated enhanced drug penetration while maintaining biocompatibility, with molecular dynamics simulations revealing that self-assembly was adjusted by noncovalent intermolecular forces [443]. Furthermore, cyclodextrin-based metal–organic framework nanosheets (CL-NS-MOF) presented significantly improved precorneal residence time and intraocular bioavailability compared to conventional formulations, suggesting that particle geometry plays a crucial role in biodistribution [444].

These integrated approaches combining rational formulation design with 3D model validation represent a paradigm shift in natural product drug development, enabling the optimization of both penetration and immunomodulatory activity essential for clinical translation.

### 6.2. Future Research Priorities

#### 6.2.1. Integration of Artificial Intelligence and Machine Learning

The complexity of optimizing formulations for both penetration and immunomodulatory activity creates opportunities for artificial intelligence (AI)-driven approaches. Machine learning algorithms have proven significant potential in addressing the penetration barriers identified in tumor microenvironments (Section 2.2) while maintaining drug activity (Section 4.1). Recent studies indicate that machine learning (ML) models can efficiently predict absorption, distribution, metabolism, excretion, and toxicity (ADMET) properties of compounds, with multi-objective optimization algorithms such as Particle Swarm Optimization (PSO) enhancing both biological activity and ADMET properties simultaneously [445]. These foundational capabilities position AI as a promising tool for addressing the penetration–activity trade-off identified in Section 4.

Deep learning models directly address the formulation stability challenges outlined in Section 4. A 12-layer deep neural network attained 73% accuracy for disintegration time and 99% for tablet hardness prediction, surpassing alternative techniques [446]. These capabilities enable precise control over drug release profiles while maintaining structural integrity, crucial for natural product formulations. These predictive capabilities extend beyond traditional formulation parameters to complex biological systems, where AI demonstrates equal promise in addressing the penetration–activity trade-off challenges. For practical implementation, recent studies demonstrate specific workflows: Schurr et al. employed a two-stage approach using Mask R-CNN for initial detection followed by U-Net++ for precise segmentation, achieving Dice coefficients of 0.8829 for fully formed spheroids [447]. This workflow typically involves: (1) training on annotated spheroid images, (2) feature extraction through convolutional layers, (3) optimization using evolutionary algorithms with mutation rates of 0.1, and (4) classification into developmental stages (monolayer, aggregating, fully formed). The SpheroidPicker system implements convolutional neural networks to achieve 97% sorting accuracy [448], while U-Net architectures enable accurate segmentation of complex 3D structures [449], establishing the technical foundation for AI-guided formulation optimization.

For the macromolecular barriers in 3D tumor models (Section 2.2.2), AI-enhanced confocal microscopy enables quantitative analysis of drug penetration into spheroids. The extracellular matrix presents physical barriers that limit drug diffusion, making accurate penetration prediction essential [448]. Machine learning approaches applied to these complex systems produced correlation coefficients of 0.963, illustrating robust prediction of drug distribution patterns [449]. High-throughput imaging combined with deep learning reduces 3D image acquisition time while maintaining quality [450], enabling real-time monitoring of formulation performance—a critical capability for optimizing the penetration–activity balance.

Natural product-based formulations benefit from specialized machine learning models tailored to their unique chemical diversity. The STarFish model, combining K-nearest neighbors (KNN), random forests, and multilayer perceptrons, achieved obtained area under the receiver operating characteristic curve (AUROC) scores of 0.910 on natural product datasets [451]. Transfer learning approaches proved particularly valuable, as shown in biological systems where adapted models doubled yield through accurate prediction [452]. The chemical complexity of natural products, combined with the spatial heterogeneity of 3D models discussed in Section 5, creates an ideal scenario for AI optimization where traditional approaches struggle.

The integration of ML with formulation optimization addresses the multi-parameter challenges presented in Section 3. Neural networks predicting drug concentrations delivered 93.44% accuracy, outperforming traditional methods by 7.5% [351]. Ensemble learning frameworks combining genetic algorithms generated coefficient of determination (R^2^) of 0.8209 for drug response prediction [453]. In practical applications, ML-driven optimization of biomaterial scaffolds yielded R^2^ values of 0.98 for training and 0.94 for test sets [454]. While these successes have not yet been specifically combined for natural product formulation in 3D spheroids, the convergence of these technologies is imminent. The regulatory acceptance of 3D models (Section 6.2.1) and the availability of standardized protocols (Section 7.1) create the infrastructure needed for AI-driven optimization.

Transfer learning significantly accelerates development timelines. In biological interface applications, transfer learning reduced calibration time while improving classification accuracy by over 4% with limited training data [455]. Models reached mean absolute error (MAE) of 8.54 months with R^2^ of 0.929, revealing precise predictive capabilities [456]. These computational approaches, when applied to the standardized 3D spheroid platforms described in Section 7.1, could enable rapid exploration of the formulation space while maintaining the critical balance between enhanced penetration and preserved bioactivity. The integration of AI with established 3D models represents the next logical step in addressing the fundamental challenges identified throughout this review, transforming the penetration–activity trade-off from a barrier into an optimization target.

Furthermore, comprehensive frameworks for AI implementation in personalized medicine are emerging, with applications ranging from patient-specific drug formulation to predictive modeling of therapeutic outcomes [457]. These advances are particularly relevant for natural product optimization, where individual metabolic variations significantly impact bioavailability and efficacy.

#### 6.2.2. Personalized Formulation Strategies

The heterogeneity observed across patient-derived organoids motivates development of personalized formulation approaches. Genomic profiling of organoids uncovers ECM gene signatures that predict optimal formulation strategies [363,458]. Recent studies demonstrate that intestinal ECM scaffolds significantly enhance differentiation marker expression, with mucin 2 (MUC2) and villin (VILL) showing markedly higher expression in organoids cultured in tissue-specific ECM compared to Matrigel [363]. This ECM-dependent expression pattern correlates with formulation requirements, as organoids with high collagen type I alpha 1 chain (COL1A1) expression displayed differential therapeutic responses when treated with liposomal versus free drug formulations [458]. Specifically, prostate cancer organoids cultured in Arg-Glu-Asp-Val (REDV)-functionalized hydrogels showed reduced actin symmetry under enhancer of zeste homolog 2 (EZH2) inhibition, illustrating ECM-dependent drug responses [458].

Functional profiling adds another personalization dimension, with immune cell composition in organoids guiding formulation selection [362,459,460]. An automated organoid platform established that patient-derived organoids maintained greater than 80% accuracy in predicting clinical responses among 21 patients investigated, with organoid size uniformity attained through monodisperse Matrigel droplet templating (400-μm organoids within 1 week) [362]. The standardized production of 1500 cells per droplet ensures statistical representation of tumor heterogeneity while maintaining reproducibility [362]. Alternative platforms using microfluidic systems have produced similar success rates, generating uniform organoids for high-throughput screening [461,462].

Tumor microenvironment modeling reveals critical formulation parameters [463,464,465]. Studies using 3D organoid models confirm that nanoparticle size significantly impacts tumor penetration, with particles smaller than 20 nm exhibiting enhanced penetration into hypoxic tumor regions [463]. For macrophage-rich tumors, particle size between 1–6 μm optimizes phagocytic uptake, with PLGA microparticles of 3–5 μm achieving encapsulation efficiencies of 89.6% and sustained intracellular retention for 48 h [464]. Additional studies verify that macrophage phagocytosis capacity varies significantly with particle properties, influencing therapeutic delivery strategies [466,467]. T cell-predominant models require different approaches, as T cell-engaging tumor organoid platforms showed enhanced CD8+ T cell activation with increased interferon-gamma (IFN-γ) and granzyme B (GZMB) expression when treated with epigenetic inhibitors [460].

Hyaluronan metabolism profiling provides actionable formulation guidance across multiple cancer types [468,469,470]. The hyaluronan activated-metabolism phenotype (HAMP+), characterized by overexpression of hyaluronan synthase (HAS) 2, HAS3, HYAL1, and KIAA1199, was identified in 20% of cell lines and 25% of patient tissues, correlating with shorter survival (*p* = 0.049) [468]. HAMP+ organoids exhibited enhanced susceptibility to combination treatment with 4-methylumbelliferone (4-MU) (HA synthesis inhibitor) and dextran sulfate (HA degradation inhibitor), providing a specific formulation strategy for this subset [468]. Treatment with PEGylated hyaluronidase (PEGPH20) in organoid models resulted in tumor-specific ECM degradation, with volume transfer coefficient (Ktrans) values showing enhanced perfusion 3 h post-treatment [469]. The cell surface hyaluronidase transmembrane protein 2 (TMEM2) has also emerged as a target for modulating ECM barriers [471].

High-throughput screening platforms enable rapid formulation optimization using diverse methodologies [461,462,472,473]. Using microfluidic systems, organoid drug testing can be completed within 7–14 days, with automated imaging capturing responses at 24, 48, and 72-h intervals [472]. Drug sensitivity testing across concentration ranges (0.2–50 μM for chemotherapeutics, 0.01–100 nM for targeted agents) uncovered that 72-h endpoints provided optimal predictive value [472]. Micro-organospheres (MOS) generated through droplet microfluidics could predict 3/4 clinically responding patients and 3/4 non-responding patients within 14 days [461]. Alternative approaches using nested array chips and mini-ring formats have shown similar predictive capabilities [473,474].

Clinical implementation validates feasibility and impact across multiple cancer types [340,346,475]. Among colorectal cancer patients, organoid-guided therapy delivered 68% positive predictive value and 78% negative predictive value, outperforming empirically guided treatment selection [340]. Integration with standard pathology workflows allows organoid establishment from surgical specimens or biopsies, with successful culture rates of 70–80% for most tumor types [340,346]. The mini-ring platform reduces reagent consumption 10-fold while maintaining assay reliability, addressing cost-effectiveness concerns [474]. Cryopreservation protocols enable biobanking of organoids for longitudinal studies and repeated testing [476]. Similar success has been reported in pancreatic, ovarian, and bladder cancers [475,477].

Regulatory considerations are addressed through standardized protocols and quality control measures [345,362,478]. The use of chemically defined matrices and serum-free culture conditions facilitates Good Manufacturing Practice (GMP) compliance, while automated liquid handling ensures reproducibility across clinical sites [362]. Integration of organoid testing into existing precision medicine frameworks, similar to genomic profiling, provides a pathway for clinical adoption [345]. Recent studies have proven the feasibility of organoid-based clinical trials, paving the way for regulatory approval [478]. The combination of ECM profiling, immune characterization, and drug sensitivity testing in patient-derived organoids represents a comprehensive approach to formulation personalization, directly addressing the penetration–activity trade-off through patient-specific optimization.

## 7. Recommendations for Standardization and Implementation

### 7.1. Proposed Standardization Framework

#### 7.1.1. Types of Stimuli and Their Biological Relevance

The tumor microenvironment presents unique physiological and biochemical characteristics that can be exploited for targeted drug delivery. Among these, pH gradients represent one of the most widely utilized stimuli. Solid tumors exhibit a pH of 6.5–6.8, significantly lower than the physiological pH of 7.4 in normal tissues [479,480]. This acidic environment results from increased glycolytic metabolism and poor perfusion in rapidly growing tumors, leading to accumulation of lactic acid [481,482]. The pH gradient becomes even more pronounced within intracellular compartments, where endosomes and lysosomes maintain pH values of 4.5–5.5 [483,484]. These pH differences enable the design of nanocarriers that remain stable during circulation but trigger drug release upon entering the acidic tumor environment. For instance, pH-responsive nanoparticles exhibited 67.5% doxorubicin release at pH 5.5 compared to only 27.0% at pH 7.4 after 48 h, highlighting the selectivity achievable through pH-responsive design [483].

Enzymatic overexpression in tumors provides another powerful stimulus for controlled drug release. MMPs, particularly MMP-2 and MMP-9, are significantly upregulated in various cancers, with expression levels 2–10 fold higher than normal tissues [485,486]. MMP-2 exists as a 62 kDa active form in tumor tissues, while MMP-9 shows variable expression patterns across different cancer types [485]. MMP-7 has been found to be overexpressed in pancreatic, colon, and breast cancers [487]. Additionally, cathepsin B, a lysosomal cysteine protease, exhibits optimal activity at pH 3.8–5.0 and is overexpressed in invasive cancers [487]. Cathepsin B specifically cleaves Gly-Phe-Leu-Gly (GFLG) sequences, while MMP-2 cleaves Pro-Leu-Gly-Val-Arg (PLGVR) sequences, enabling the design of enzyme-responsive drug delivery systems that release their payload specifically in the tumor microenvironment [487,488].

Oxidative stress represents another hallmark of cancer that can be exploited for drug delivery. Tumor tissues exhibit reactive oxygen species (ROS) levels of approximately 10^−4^ M, which is 5000-fold higher than the 2 × 10^−8^ molar (M) found in normal tissues [489,490]. This dramatic difference in ROS concentration enables the use of ROS-sensitive linkers such as thioketal bonds, which remain stable under normal physiological conditions but undergo rapid cleavage in the presence of elevated ROS. Studies have revealed that thioketal-containing nanoparticles can achieve 76% drug release within one hour in the presence of hydrogen peroxide [489], while other ROS-responsive systems utilizing similar thioketal chemistry have shown comparable rapid release kinetics [479,491].

The differential expression of glutathione between extracellular and intracellular environments provides yet another mechanism for controlled drug release. Intracellular glutathione (GSH) concentrations reach 10 mM, which is 500–5000 times higher than the extracellular concentration of 2–20 μM [492]. This gradient is particularly useful for designing disulfide-containing drug carriers that remain stable in circulation but undergo rapid reduction and drug release upon cellular internalization. The GSH-mediated reduction follows predictable kinetics, with complete cleavage achievable within hours of cellular uptake [492,493].

Temperature represents an externally controllable stimulus that can be combined with endogenous triggers for enhanced specificity. Thermosensitive polymers such as poly (N-isopropylacrylamide) (PNIPAM) exhibit a lower critical solution temperature (LCST) of 32–34 °C, below body temperature [484]. When combined with mild hyperthermia (41–43 °C), these systems can achieve rapid drug release. Low-temperature sensitive liposomes have achieved 90% doxorubicin (DOX) release within 5 min at 42 °C, compared to minimal release at 37 °C [484,494]. This approach has proven particular promise in clinical applications, with several thermosensitive formulations currently in clinical trials.

The integration of multiple stimuli-responsive mechanisms offers opportunities for enhanced specificity and control. Dual pH- and ROS-responsive systems have delivered superior performance compared to single-stimulus systems, achieving more precise spatiotemporal control over drug release [483,489]. Similarly, combinations of enzyme-cleavable linkers with pH-sensitive components have produced synergistic effects, with sequential activation mechanisms that minimize off-target drug release while maximizing therapeutic efficacy in the tumor microenvironment [487,488].

These biological stimuli have been successfully translated into clinical applications. FDA-approved formulations such as Doxil and Onivyde utilize pH gradients for drug loading and release, while ONPATTRO represents the first approved RNA interference (RNAi) therapeutic utilizing stimuli-responsive delivery [495]. The clinical success of these formulations validates the therapeutic potential of stimuli-responsive drug delivery systems and provides a foundation for the development of next-generation responsive therapeutics.

#### 7.1.2. Imaging Specifications and Analysis Parameters

For accurate drug penetration assessment in tumor spheroids, standardized imaging and analysis parameters are essential. Confocal microscopy with z-stack acquisition enables 3D visualization of drug distribution throughout the spheroid structure. Z-stack images should be acquired at 10 μm intervals throughout the spheroid volume to capture complete 3D drug distribution patterns [496]. This interval provides sufficient spatial resolution while maintaining practical data acquisition times for high-throughput screening applications.

When using fluorescent drug conjugates or tracers, validation against the parent compound is critical for accurate penetration assessment. The correlation between fluorescent tracer and parent drug distribution should exceed 95% to ensure that the fluorescent modification does not significantly alter drug penetration behavior [496]. This validation can be performed using MALDI-MSI or liquid chromatography-tandem mass spectrometry (LC-MS/MS) techniques, which enable direct comparison of drug and metabolite localization within spheroid sections [497,498].

Temporal analysis of drug penetration requires assessment at multiple time points to capture both initial distribution and steady-state accumulation. Standard imaging time points of 1, 24, and 72 h post-treatment enable characterization of penetration kinetics, with 24 h representing clinically relevant drug exposure duration [499,500]. For continuous perfusion studies, flow rates of 19–96 μm/s approximate physiological interstitial flow conditions and influence drug penetration patterns [323].

Radial profiling analysis quantifies drug distribution from the spheroid periphery to the core using concentric bins of 10 μm width for high-resolution analysis [501]. The penetration depth is typically defined as the distance at which drug concentration falls to 50% of the peripheral concentration [442]. This standardized metric enables comparison across different drugs and spheroid models.

For statistically robust analysis, experiments should include 3–7 biological replicates per condition, with spheroids of consistent size and morphology [502]. Spheroids exceeding 500 μm in diameter develop the characteristic three-layer structure (proliferating rim, quiescent zone, and necrotic core) that mimics solid tumor microenvironments and creates physiologically relevant diffusion barriers [503]. The formation of these gradients is essential for modeling drug penetration barriers observed in solid tumors [503].

Drug distribution can be validated using MALDI-MSI, which detects both parent drugs and metabolites with specific mass-to-charge ratio (*m*/*z*) values (e.g., irinotecan at *m*/*z* 587 and its active metabolite SN-38 at *m*/*z* 393) [497]. Measurements at 24 and 48 h uncover drug accumulation primarily at the spheroid periphery, with limited penetration to the core regions [497]. Quantitative validation can employ tissue extinction coefficients compared with LC-MS/MS data to account for ion suppression effects in different tissue regions [504]. This spatial-temporal data provides mechanistic insights into penetration barriers and metabolism patterns essential for optimizing drug delivery strategies.

#### 7.1.3. Integration with Immune Cell Co-Culture Systems

The established spheroid models were utilized to investigate tumor-immune cell interactions, a critical component of the tumor microenvironment [268,277,505]. Spheroids were formed using hanging drop methods or ultra-low attachment plates within 24–48 h and maintained for extended periods up to 72 h, with viability confirmed by live/dead staining at 24, 48, and 72-h intervals [506,507].

For T cell-tumor interactions, spheroids were co-cultured with primary T cells at a 1:1 ratio, which served as a predictor of cytotoxic efficiency [277]. T cell activation was achieved using anti-CD3/CD28 antibodies at 10 μg/mL with a 2:1 mass ratio of CD3:CD28, demonstrating superior expansion compared to other tested ratios [508]. Following 72 h of co-culture, T cell activation was confirmed by flow cytometric analysis of CD69 (early activation) and CD25 (late activation) markers [509].

Activated T cells exhibited distinct infiltration patterns, with cells migrating as either single cells or clusters within spheroids [277]. Notably, spheroid size significantly affected cytotoxic efficiency, with larger spheroids displaying higher hypoxia levels and reduced T cell activation characterized by decreased IFN-γ, TNF-α, and granzyme B expression [277]. Spatial analysis uncovered lower T cell numbers and cytotoxicity in the spheroid core compared to the periphery, highlighting the importance of the 3D architecture [277,510].

For macrophage studies, spheroids were co-cultured with macrophages at a 3:1 tumor:macrophage ratio using transwell systems or direct co-culture methods [271,505]. Macrophage polarization was induced using established protocols: M1 polarization with IFN-γ (10 ng/mL) combined with lipopolysaccharide (LPS) for 24 h, while M2 polarization utilized IL-4 treatment for the same duration [511,512]. Successful polarization was confirmed through surface marker expression patterns, with M1 macrophages presenting CD80hi/CD206lo phenotype and M2 macrophages showing CD80lo/CD206hi expression [511,513].

Temporal analysis of immune cell infiltration indicated that the first CD11b+ cells were detected at 18 h post-co-culture, with inflammatory marker expression peaking at 24 h before sharply attenuating by 3 days [514]. Functional characterization included cytokine profiling, with TNF-α and IL-6 levels measured at 6-h intervals, revealing time-dependent secretion patterns [512,514]. Additionally, reactive oxygen species generation served as a functional indicator of M1 macrophage activation, while phagocytic activity was assessed to evaluate macrophage function [512,513].

Spatial distribution analysis using immunofluorescence microscopy exposed differential localization patterns, with CD206+ M2 macrophages preferentially accumulating at the tumor invasive front (5.23%) compared to the tumor interior (2.59%) [515]. This distribution pattern was consistent with in vivo observations and emphasized the model’s ability to recapitulate physiologically relevant immune cell behaviors [512,515]. The integrated platform illustrated the complex interplay between tumor cells and immune components, providing a valuable system for evaluating immunotherapeutic strategies in a controlled 3D microenvironment [33,277,505].

### 7.2. Implementation Strategies

#### 7.2.1. Nanomedicine-Based Delivery Systems

Nanomedicine approaches have shown significant promise in enhancing the tumor penetration of polysaccharide immunomodulators. These systems leverage various strategies including liposomal formulations, polymeric nanoparticles, and surface modifications to overcome the barriers of tumor microenvironment.

Liposomal delivery systems have yielded remarkable improvements in drug penetration into tumor spheroids. Studies have shown that the penetration efficiency of liposomes strongly correlates with their membrane rigidity, with a correlation coefficient of 0.84 between bending modulus and spheroid penetration [12]. Curcumin-loaded deformable liposomes attained enhanced penetration into tumor models, with improvements ranging from 1.78 to 266-fold depending on formulation [516]. The incorporation of PEGylation further enhanced penetration, with PEGylated liposomes displaying significantly improved accumulation in spheroid cores within 4 h [11].

Polymeric nanoparticle systems offer tunable properties for optimized delivery. PLGA-based nanoparticles conjugated with cell-penetrating peptides exhibited size-dependent penetration, where 20 nm particles achieved 10-fold higher uptake compared to 100 nm particles [517]. Interestingly, the drug release kinetics also played a crucial role, with low drug content conjugates (20 nm) showing faster release rates of 5%/day compared to 1–3%/day for high drug content conjugates (30–120 nm), resulting in superior tumor penetration and therapeutic efficacy [195].

Surface modification strategies have proven particularly effective for enhancing both penetration and bioactivity retention. Polysaccharide conjugation to TLR7 ligands increased their immunostimulatory potency by 10–1000 fold compared to unconjugated ligands [518]. Cell-penetrating peptides such as R8-dGR enabled efficient penetration into glioma spheroids while maintaining biological activity [519]. Moreover, MMP-2 sensitive systems illustrated selective activation in the tumor microenvironment, with masked cell-penetrating peptide (CPP)-drug conjugates showing enhanced tumor specificity [520].

The incorporation of stimuli-responsive elements has further improved delivery efficiency. Nitric oxide (NO)-driven nanomotors promoted not only their own penetration but also enhanced T cell infiltration from 2.1% to 28.2% in tumor tissue [521]. Similarly, surface acoustic wave (SAW) treatment improved nanoparticle distribution throughout spheroids, overcoming the typical limitation of peripheral accumulation [522].

#### 7.2.2. Roadmap for Clinical Translation

**Figure 6:**
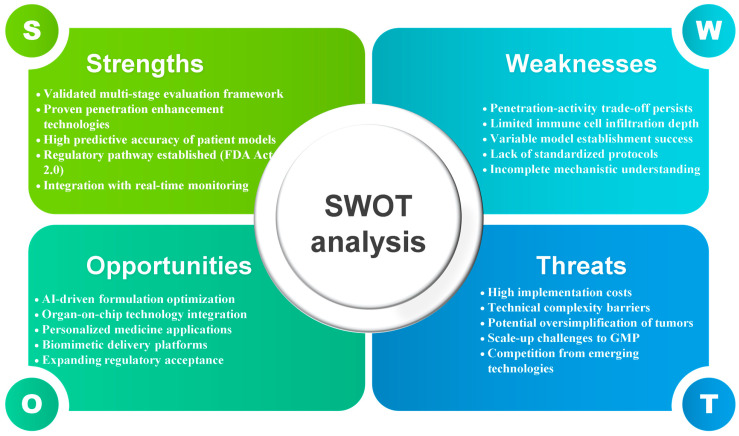
Strengths, weaknesses, opportunities, threats (SWOT) analysis of natural product formulation in 3D tumor models.

Successful translation requires phased development acknowledging both scientific and regulatory requirements. The integration of 3D models with clinical practice necessitates a systematic approach that bridges laboratory innovation with patient care (Figure 6).

Phase 1 focuses on standardized platform validation, establishing reference formulations with documented performance across multiple laboratories. Recent advances in automated systems illustrate the feasibility of high-throughput production, with platforms achieving over 50 uniform spheroids with consistent size and circularity in 30 min with over 97% sorting accuracy [436]. Achieving reproducible results across laboratories requires stringent quality control, as coefficient of variation (CV) values less than 10% are evaluated as good cell-based assays [523]. The implementation of Quality by Design (QbD) frameworks, successfully applied in pharmaceutical 3D printing, provides a model for systematic quality control in 3D culture systems [524]. This standardization approach has been validated through automated spheroid production platforms achieving CV values below 3% and 97% sorting accuracy within 30 min, demonstrating the feasibility of high-throughput natural product screening with reproducible results across multiple laboratories [436].

Phase 2 incorporates patient-derived organoids, correlating in vitro penetration–activity relationships with clinical markers. Large-scale validation studies reveal that PDO drug screening attains 68% positive predictive value and 78% negative predictive value for standard chemotherapy responses [525]. Machine learning approaches applied to PDO data reach 68.75% accuracy with random forest classifiers in predicting drug responses [526]. Establishment success rates vary significantly across cancer types, with PDO generation successful in 71% of pancreatic cancer patients who had received neoadjuvant therapy [525], while ovarian cancer organoid derivation accomplished 36% for high-grade serous ovarian cancer patients and 44% for all ovarian cancer patients combined [527], highlighting the importance of cancer-specific protocol optimization. The clinical relevance of this approach is demonstrated by patient-derived organoid studies achieving 68% positive predictive value and 78% negative predictive value for treatment responses, with machine learning integration reaching 68.75% accuracy in predicting drug responses across colorectal and bladder cancer patients [344].

Phase 3 emphasizes early clinical translation through window-of-opportunity trials in surgical patients. The Phase Ia trial of Boswellia serrata in breast cancer patients exemplifies this approach, where pre-operative administration until the night before surgery allowed correlation of ex vivo tissue analysis with drug effects [528]. Ki-67 proliferation markers served as primary endpoints, showing a statistically significant reduction in proliferation between core biopsy and excision in the treatment group (13.8 ± 11.7% reduction) compared to control (54.6 ± 21.4% increase, *p* = 0.008) [528]. Building on this window-of-opportunity concept, novel approaches include implantable microdevices that enable testing up to 20 therapies simultaneously within patient tumors, with drug effects examined within days rather than months [433]. These real-time assessment capabilities are further enhanced by advances in intraoperative imaging technologies, which establish that in vivo assessment produces higher sensitivity and negative predictive value than ex vivo analysis [529]. This design enables validation of 3D model predictions against actual patient tissue responses. This strategy is exemplified by natural product window-of-opportunity trials, where Boswellia serrata administration in breast cancer patients demonstrated statistically significant Ki-67 proliferation marker reduction (13.8% reduction vs. 54.6% increase in control, *p* = 0.008), validating 3D model predictions through direct tissue analysis [528].

Phase 4 scales successful formulations through GMP manufacturing, with quality specifications based on 3D model performance rather than traditional pharmaceutical metrics. The development of GMP-compliant protocols for organoid production, exemplified by replacing R-spondin-1 conditioned medium with recombinant protein, confirms the feasibility of standardized manufacturing [530]. Adaptive clinical trial designs incorporating organoid-based interim analyses enable rapid optimization, as evidenced by trials where organoid-guided therapy outperformed empirically guided treatment selection [525] Successful implementation is demonstrated by GMP-compliant organoid production protocols that replaced conditioned media with recombinant proteins, enabling standardized manufacturing while maintaining organoid-guided therapy performance that outperformed empirically guided treatment selection in clinical validation studies [340].

## 8. Conclusions

This comprehensive review unveils the complex landscape of natural product formulation for 3D tumor models, highlighting both remarkable technological advances and critical gaps in translating enhanced penetration into preserved immunomodulatory activity. Our analysis establishes that while sophisticated formulation strategies achieve impressive penetration enhancements—ranging from 3- to 20-fold across different natural product categories—a fundamental disconnect exists between improved delivery and biological efficacy. The penetration–activity trade-off emerges as the central challenge. Enhanced penetration frequently occurs at the expense of biological activity through multiple mechanisms including conformational changes during encapsulation, incomplete drug release, and surface modifications that interfere with molecular recognition. This trade-off is exemplified by curcumin, where EC_50_ values increase from 12.25 μM in 2D ultures to 30.76 μM in 3D spheroids despite formulation enhancements achieving deep tissue penetration. Moving forward, success requires integrated assessment frameworks evaluating both penetration and immunomodulatory preservation. Table 3 presents a comprehensive framework that integrates all aspects discussed in this review, serving as a practical guide for researchers navigating this complex landscape. Finally, to provide strategic guidance for implementation, we present a strengths, weaknesses, opportunities, threats (SWOT) analysis (Figure 6) that synthesizes the technical considerations into an actionable framework for stakeholders. The convergence of advanced formulation technologies, sophisticated 3D culture systems, and regulatory support through FDA Modernization Act 2.0 positions the field for transformative advances. Only through recognition that penetration without activity preservation is meaningless can we deliver effective natural product-based cancer immunotherapeutics.

## Figures and Tables

**Table 2:** Natural product categories and formulation requirements based on physicochemical challenges affecting 3D tumor penetration. Data compiled from literature (2012–2025) showing preferred strategies and reported outcomes. Arrow (→) indicates change from 2D to 3D culture conditions. 2D, two-dimensional; 3D, three-dimensional; EC_50_, half maximal effective concentration; EGCG, epigallocatechin gallate; kDa, kilodalton; NPs, nanoparticles; pH, potential of hydrogen; PSK, polysaccharide-K; t½, half-life; μm, micrometer.

Category	Representative Compounds	Primary Challenge	Preferred Formulation Strategy	Reported Outcomes
Hydrophobic	Curcumin Resveratrol EGCG Quercetin	Poor solubility; Protein binding	Nanoparticles Liposomes Cyclodextrin	Enhanced penetration (3–20 fold) with nanoformulations; Reduced efficacy in 3D vs. 2D (EC_50_: 12.25 → 30.76 μM for curcumin) [11,12,60]
Macromolecular	Lentinan PSK Fucoidan	Size exclusion (>10 kDa)	Surface modification Enzyme-responsive systems	Limited penetration (30–50 μm) even with modifications; Size-dependent exclusion [11,66]
Stability -sensitive	Anthocyanins Catechins (including EGCG)	Environmental degradation	pH-responsive coating Antioxidant encapsulation	Anthocyanins: t½ = 1.98 h (pH 7.0, 75 °C) vs. 15.12 h (pH 3.0, 75 °C) [76]; EGCG: t½ < 30 min in culture media [79]

**Table 3:** Integrated framework for overcoming the penetration–activity trade-off in 3D tumor models. Overview of challenges and solutions across four domains: tumor microenvironment barriers, natural product categories, 3D model evolution, and translation framework. Framework synthesizes current achievements with strategic solutions for developing effective natural product-based cancer immunotherapies. Upward arrow (↑) indicates fold-increase or enhancement; ≠ symbol indicates "not equal to" or lack of correlation. 3D, three-dimensional; CV, coefficient of variation; ECM, extracellular matrix; EC_50_, half maximal effective concentration; EGCG, epigallocatechin gallate; FDA, Food and Drug Administration; GMP, Good Manufacturing Practice; kDa, kilodalton; MW, molecular weight; NPV, negative predictive value; PPV, positive predictive value.

Category	Subcategory	Barriers/Challenges	Current Achievements	Strategic Solutions	Clinical Implications
I. Tumor Microenvironment	Physical barriers	ECM density (33-fold ↑ fibronectin) [38,39,40]; Cell density (6 × 10^7^ cells/cm^3^) [45,46]; Tortuous diffusion pathways	Size-dependent penetration; <50 nm particles reach core; >100 nm remain peripheral [11,94]	Matrix-modifying enzymes; Ultrasound (6–20 fold ↑) [162,163]; Hyperthermia (38.3–45 °C) [220,221]	Temporal modification windows; Safety vs. efficacy balance
	Chemical gradients	pH (7.4 → 6.5 tumor; 4.5–5.5 lysosomes) [54,55]; O_2_ (<0.2% in core) [50,51]; Nutrients/metabolites	pH-responsive release (87.4% at pH 6.5) [192]; Hypoxia-activated prodrugs; ROS-responsive systems	Multi-stimuli responsive design; Sequential release systems; Gradient exploitation	Zone-specific therapy; Reduced off-target effects
	Biological factors	Immune cells at periphery (30–50 μm) [268,269,272]; Drug efflux pumps (CYP3A4 200-fold ↑) [343]; Metabolizing enzymes	Macrophage transport (2–5× deeper) [270]; NO nanomotors (T cells: 2.1 → 28.2%) [521]; TAM reprogramming	Biomimetic carriers; Cell membrane coating; Immunomodulation	Focus on immune-active zones; Combination strategies
II. Natural Product Categories	Hydrophobic	Poor solubility (curcumin: 0.6–7.8 μg/mL) [57,58,59]; >95% protein binding [61,62]; Aggregation	3–20 fold penetration ↑ [94,231,232]; EC_50_: 12.25 → 30.76 μM (2D → 3D) [60]; Enhanced delivery achieved	Nanoparticles (PLGA, SLN); Liposomes; Cyclodextrins (206-fold ↑) [104]	Activity-penetration trade-off; Formulation-dependent efficacy
	Macromolecular	Size exclusion (>10 kDa) [11,66]; Limited to 30–50 μm [11,66]; MW-activity relationship	Surface modifications tested; Enzyme conjugation developed	Peripheral targeting; Matrix modification; Ultrasound delivery	Preserve MW-dependent activity; Alternative delivery paradigms
	Stability-sensitive	pH degradation; Oxidation (EGCG t½ < 30 min) [79]; Temperature sensitivity	Protected formulations developed; Sustained activity achieved	pH-responsive coatings; Antioxidant co-encapsulation; Solid lipid matrices	Maintain bioactivity; Controlled release
III. Three-dimensional Model Evolution	Spheroids	Size/composition variability; Drug resistance (IC_50_ varies 160%) [353]	CV < 3% with automation [435,436]; Reproducible protocols	Standardized methods; Co-culture integration	High-throughput screening; Predictive models
	Organoids	Establishment (20–85.7%) [349,350]; Patient heterogeneity	68% PPV, 78% NPV [340]; 97% mutation retention [362]	Patient-specific matrices; Alternative scaffolds	Personalized medicine; Biomarker discovery
	Advanced systems	Vascularization complexity; Dynamic flow; Multi-organ interactions	Real-time monitoring; First-pass metabolism revealed	Microfluidic platforms; Organ-on-chip	Physiological relevance; Systems pharmacology
IV. Translation Framework	Assessment	Lack of integrated metrics; Penetration ≠ efficacy	5-stage framework developed [Section 4.2.3]; Spatial-functional analysis	Multi-modal imaging; Zone-specific evaluation	Holistic optimization; Activity preservation
	Regulatory	No specific guidelines; Inter-country differences	FDA Modernization Act 2.0 [352]; 3D model acceptance	Harmonized standards; QbD implementation	Accelerated approval; Reduced animal testing
	Implementation	Scale-up challenges; Cost considerations	Automated production (97% accuracy) [436]; GMP protocols	Phase 1–4 roadmap; Adaptive trials	Clinical translation; Market readiness

## Data Availability

Data are contained within the article.
